# SARS-CoV-2 Pandemic Tracing in Italy Highlights Lineages with Mutational Burden in Growing Subsets

**DOI:** 10.3390/ijms23084155

**Published:** 2022-04-09

**Authors:** Angelo Boccia, Rossella Tufano, Veronica Ferrucci, Leandra Sepe, Martina Bianchi, Stefano Pascarella, Massimo Zollo, Giovanni Paolella

**Affiliations:** 1 Ceinge Biotecnologie Avanzate, 80145 Naples, Italy; boccia@ceinge.unina.it (A.B.); tufano@ceinge.unina.it (R.T.); veronica.ferrucci@unina.it (V.F.); leandra.sepe@unina.it (L.S.); 2 Dipartimento di Medicina Molecolare e Biotecnologie Mediche (DMMBM), Università degli Studi di Napoli Federico II, 80131 Naples, Italy; 3 Department of Biochemical Sciences “A. Rossi Fanelli”, Sapienza Università di Roma, 00185 Rome, Italy; martina.bianchi@uniroma1.it (M.B.); stefano.pascarella@uniroma1.it (S.P.); 4 DAI Medicina di Laboratorio e Trasfusionale, AOU Azienda Ospedaliera ‘Federico II’, 80131 Naples, Italy

**Keywords:** SARS-CoV-2, variant of concern (VOC), viral variants tracing

## Abstract

Tracing the appearance and evolution of virus variants is essential in the management of the COVID-19 pandemic. Here, we focus on SARS-CoV-2 spread in Italian patients by using viral sequences deposited in public databases and a tracing procedure which is used to monitor the evolution of the pandemic and detect the spreading, within the infected population of emergent sub-clades with a potential positive selection. Analyses of a collection of monthly samples focused on Italy highlighted the appearance and evolution of all the main viral sub-trees emerging at the end of the first year of the pandemic. It also identified additional expanding subpopulations which spread during the second year (i.e., 2021). Three-dimensional (3D) modelling of the main amino acid changes in mutated viral proteins, including ORF1ab (nsp3, nsp4, 2’-o-ribose methyltransferase, nsp6, helicase, nsp12 [RdRp]), N, ORF3a, ORF8, and spike proteins, shows the potential of the analysed structural variations to result in epistatic modulation and positive/negative selection pressure. These analyzes will be of importance to the early identification of emerging clades, which can develop into new “variants of concern” (i.e., VOC). These analyses and settings will also help SARS-CoV-2 coronet genomic centers in other countries to trace emerging worldwide variants.

## 1. Introduction

SARS-CoV-2 is a human coronavirus (CoV) responsible for the coronavirus disease 19 (COVID-19) pandemic. Human coronaviruses are members of the Nidovirales order and belong to Coronaviridae family. To date, seven species of human coronaviruses have been identified: HCoV-NL63, HCoV-229E, HCoV-OC43, HCoVHKU1, SARS-CoV, MERS-CoV, and SARS-CoV-2 [1]. Like other coronaviruses, SARS-CoV-2 is an enveloped virus with a positive-sense, single-stranded RNA genome. SARS-CoV-2 belongs to the genus betacoronavirus, together with SARS-CoV and MERS-CoV (with 80% and 50% homology, respectively) [2,3]. Coronaviruses (CoVs), including SARS-CoV-2, possess the largest genomes (26–32 kb) among all RNA virus families, flanked by 5′ and 3′ untranslated regions. During their viral cycle, CoVs replicate their genomic RNA (gRNA) to produce full-length negative (antisense) RNA molecules acting as the template for the synthesis of positive-sense gRNA molecules that are then packaged with the structural proteins into newly assembled virions.

All SARS-CoV-2 RNAs contain a common ‘‘leader’’ sequence (70 nt). Upon cell entry, the viral gRNA is translated to produce nonstructural proteins (nsps) from two large open reading frames (ORFs), ORF1a and ORF1b, via proteolytic cleavages [4]. Among them, 15 nsps compose the viral replication and transcription complex (RTC). Of importance, nsp12 (harboring RNA-dependent RNA polymerase, RdRP) leads replication and transcription mechanisms by using viral RNA as the template. A hallmark of CoVs is the “discontinuous transcription” mechanism, which produces a set of subgenomic RNAs (sgRNAs) following the “leader-to-body fusion” model [2]. Briefly, negative-strand synthesis by RTC is interrupted when the nascent RNA complex encounters one of the transcription regulatory sequences (TRS ‘body’, or TRS-B) found before most ORFs located within the 3′ region of the viral genome. Negative-strand RNA synthesis is then re-initiated following the interaction of its terminal TRS with a complementary positive-strand TRS (TRS ‘leader’, or TRS-L) located in the leader sequence, about 70 nucleotides from the 5′ end of the genome. Upon re-initiation of RNA synthesis at the TRS-L region, a negative-strand copy of the leader sequence is added to the nascent RNA to complete the synthesis of negative-strand sgRNAs. These fused negative-strand intermediates are then used as templates, to synthesize positive-sense sgRNAs, which are in turn translated into both structural and accessory proteins [5]. The ORFs contained within sgRNAs encode structural proteins (spike [S], envelope [E], membrane [M], and nucleocapsid [N]) and several accessory proteins (including 3a, 6, 7a, 7b, 8, and 10). Of importance, the early sgN and sgE transcripts are the first and most abundant RNA transcripts to be produced [6,7]. During initial (negative-strand) replication, the RNA genome may incorporate random mutations, mainly because of the lower activity of nsp14, a proofreading exonuclease which ensures replication competence during the expansion and maintenance of such large genomes [8]. These mutations are then copied into the nascent SARS-CoV-2 RNA genome and can be subjected to purifying selection while the virus spreads from cell to cell.

The evolution of the SARS-CoV-2 pandemic has been studied and sequence diversity has been found to steadily grow within each analyzed geographic region. It has been primarily characterized by purifying selection, with a small set of sites evolving under positive selection. Some early mutations (including S:G614D), which possibly only provide a modest selective advantage in isolation, have been shown to exert a much greater effect through multiple epistatic interactions [9]. For example, multiple positively selected sites in the S protein RBD are signature mutations in emerging variants, and some have been demonstrated to result in neutralizing antibody evasion. More surprisingly, the N protein also includes several sites that appear to be strongly selected (i.e., N:T205I, N:R203K, N:G204R, and N:G204M). Additional mutations with positive selection have been identified in ORF1a (ORF1a:3675- and ORF1a:3676-) by studying the different isolated variants (alpha, beta, gamma, eta, iota, and lambda). Some of the mutations, for which positive selection was inferred, co-occur on multiple occasions and form a strongly connected network of apparent epistatic interactions. The early events that shaped the epistatic network likely laid the foundation for virus diversification in relation to virulence, immune evasion, and transmission.

Virus spread in Italy occurred during the early stages of the epidemic in the first months of 2020 [10], preceding the diffusion to other European countries, and was initially concentrated in an area corresponding to part of northern Italy, with the south being far less affected [11,12]. This initial spread also involved areas of neighboring countries with almost no limitations until the first border blockade in March [13]. After this initial spread, the epidemic went through a resting phase in the summer, but reemerged—this time, generalized to the whole country, in the last months of 2020 [14,15,16].

The present work was focused on the spread of SARS-CoV-2 virus within the Italian infected population and uses the virus sequences deposited by the Italian sequencing centers, to build a filogenetic tree containing all sampled viral sequences. A number of expanding virus subpopulations was identified by analyzing and selecting subtrees actively growing at the end of the first year of the epidemic. A tracing procedure, based on the same tools and set up to monitor the further evolution of the epidemic, demonstrated that these subtrees also characterized the spread of the epidemics during 2021 and effectively highlighted the spread of further clades/subclades with potential positive selection within the infected population. Analysis of the sequence of such variant viruses identified mutations in spike and other fundamental viral proteins associated with the expanding subpopulations. Furthermore, molecular modelling showed that these mutations have the potential to influence protein function by altering stability or by affecting 3D structure.

## 2. Results

### 2.1. Epidemic Trend in Italy during the First Year

To investigate the trend of the SARS-CoV-2 epidemic in Italy during the first year, we performed a phylogenetic analysis on a dataset consisting of viral sequences from Italy and neighboring countries, bordering the eleven municipalities of Northern Italy hit hardest at the beginning of the pandemic and involved, before the first lockdown, in daily transfer of people for various reasons, including those work-related. The analysis was carried out by running the Nextstrain pipeline [17], with parameters described under the Materials and Methods section, on a dataset containing all the Italian sequences submitted to GISAID [18,19,20] at the time when the analysis was performed (8 January 2021), and a sample of the sequences from the neighboring countries, selected on the basis of population and spreading degree of SARS-CoV-2 infection, in order to avoid overrepresentation of sequences from countries, including Switzerland, who, following a different sequencing policy, had submitted many more sequences than others. The analyzed dataset contained 1655, 509, 294, 481, and 108 sequences from Italy, Switzerland, South Germany, Austria, and Slovenia, respectively. The dataset was completed by adding 742 sequences from the rest of the world to ensure the presence of all major globally spread virus subpopulations.

The distribution of sequences is not homogeneous during the year and the tree shows a clear reduction in Italian sequences sampled in the months immediately following the introduction of restriction measures in March, most likely a consequence of strongly decreased viral infections. This reduction confirms the effectiveness of the locking-down measures introduced to counter virus spread, as observed in other reports [14]. 

**Figure 1:**
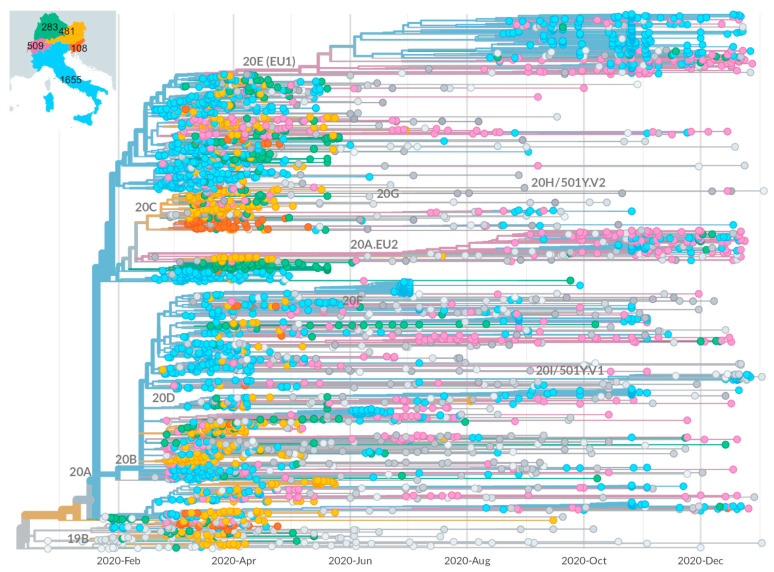
Phylogenetic analysis of the 2020 dataset. The analysis is focused on viral sequences from Italy and neighboring countries, sampled during 2020. The number of samples from each country of the focus is reported in the inset. The samples in the tree are colored on the basis of their origin, according to the colors used in the inset; the samples in gray are additional from the rest of the world, added to ensure the presence of all major globally spread virus subpopulations. The occurrence of known clades is indicated by the labels near the top node of the corresponding sub-tree. Bottom bars indicates time periods in which the Italian government implemented stringent (black) or relaxed (gray) mobility restrictions.

According to this analysis (see Figure 1), the first forms of the virus, corresponding to clades 19A and 19B, appeared at the beginning of the pandemic, but faded away by the end of March 2020. In fact, the newer clade 20A, which characterized the entry of the pandemic in Europe, was already predominant by then and soon spread throughout the globe. This variant was shown to be characterized by the presence of a mutation in the spike protein gene, D614G, which gave the virus an advantage in terms of transmissibility [21,22]. Starting from clade 20A, subtypes 20B and 20C evolved; however, while 20B widely spread to all analyzed regions, 20C was notably largely unrepresented in Italy. A few 20C isolates were detected only after the August bottleneck, in contrast with other European countries where the clade was observed as early as March and remained, although limited to about 4% of the isolates, for the rest of the year. Since August 2020, two more clades, 20E (EU1) and 20A.EU2, also derived from 20A, spread extensively and accounted for about 50% of the submitted sequences by the end of the year. This diffusion is in agreement with data from Hodcroft et al. [23], who initially observed it in Spain in the early summer, and associated its later spread with the summer lift of blockades of national borders. During December, the so-called “UK” variant 20I/501Y.V1, also known as 20I (Alpha, V1) and B.1.1.7, first identified in the UK in September 2020 [24,25], started to spread in the analyzed area, reaching about 10% of reported cases at the end of 2020.

### 2.2. Identification of Growing Virus Subsets in Italy

In order to identify growing virus subsets, corresponding to potentially new viral variants which can spread within the area, a procedure was set up to detect and characterize, within the phylogenetic tree, expanding monophyletic virus subtrees. The procedure, described in detail in the Materials and Methods section, scans the tree and assigns a score to each internal node, calculated on the basis on the number of descendants and the apparent expansion rate, this last evaluated as the skewness of the distribution of the collection dates of its child leaves. Higher scores are assigned to nodes with a larger number of descendants and more negatively skewed distribution. The procedure also filters out subsets that are either smaller than a given threshold, or larger than 5% of the total number of leaves, to avoid results which would deviate too much from the concept of an emerging subset. The same threshold is used to prevent the selection of almost identical subtrees.

**Figure 2:**
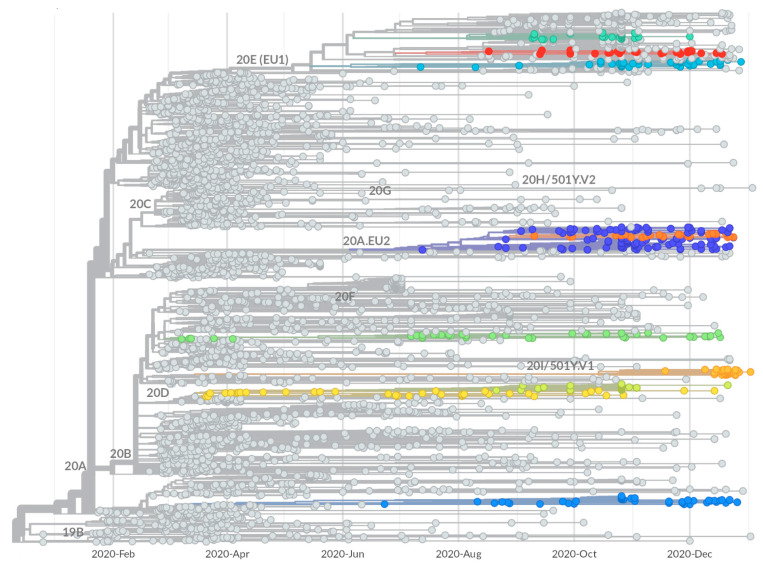
Growing subsets. The identified ten subsets are shown in the context of the phylogenetic tree, using colors. Subsets contained in a larger one are indicated by their names, separated by dash, as for 2–3, in which S2 is contained in S3. The occurrence of known clades is indicated by the labels near the top node of the corresponding sub-tree. The bottom bars indicate the time periods in which the Italian government implemented stringent (black) or relaxed (gray) mobility restrictions.

The execution of the procedure onto the described phylogenetic tree yields ten growing subtrees, displayed as colored branches in Figure 2. The isolates contained within the ten subtrees were used to produce the virus subsets (S1–S10), summarized in Table 1. For the identified subsets, the calculated scores range between 54.8 and 5.8. The highest score was obtained by the S4 subset, which is relatively small (43 viruses), and its high score is mainly due to the skew value, −3.07, which is better than that of all other subsets, and possibly indicative of a more rapid expansion. The higher scores of S3, S8, and S10 are also associated with strong skew, whereas S7 is selected thanks to the larger number of viruses combined to a lower, but still clearly negative, skew. In some cases, subsets identified in this way are sub-subsets of other subsets; we introduced the concept of a family of subsets to account for this kind of relationship, as the two families 2–3 and 6–7 are composed of the S2–S3 and S6–S7 subsets, respectively. All subsets include at least 50% sequences from Italy, except for S7 and S5, which have a majority of sequences from Switzerland, especially in the second case where they exceed 90%.

The relationship between subsets and known Nextstrain clades was assessed by evaluating the fraction of leaves shared between each subset and each clade; the results, reported in full in Table S1 in the Supplementary Materials, are used to fill the two clade columns of Table 1. Within them, ‘is’ indicates a clade which perfectly corresponds with the subset, i.e., sharing over 95% of the elements, whereas the name reported in the ‘parent’ column indicates the clade from which a given subset is derived. As reported in the “is” column”, S4 corresponds with the Nextstrain clade 20I/501Y.V1 (the “UK” variant), while S7 corresponds to the above mentioned clade 20A.EU2. The other subsets do not fully overlap with any of the known clades, but of course all derive from one. S1 derives from clade 20A; S2 and S3 are part of a family and both are included in clade 20D; S5 springs from clade 20B; S6 derives from clade 20A.EU2 and is included in S7 which fully corresponds to it; and S8, S9, and S10 are not related to each other, but all descend from 20E (EU1), with which they share between 8% and 12% of the virus isolates.

The identified subsets were further characterized by using the Pango lineage annotation available in the GISAID-derived dataset (see column Pango of Table 1). S4 and S7, previously identified as Nextstrain clades 20I/501Y.V1 and 20A.EU2, also correspond with the equivalent Pango lines B.1.1.7 and B.1.160, respectively. In addition, S1 was found to correspond with Pango B.1.258, a line described as circulating in Central Europe since August 2020 [26], while S5 corresponded with Pango B.1.1.39, a lineage mostly observed in Switzerland [27]. For the other subsets (in yellow in Table 1), the analysis failed to give further information, as they were identified as the Pango lines corresponding to the Nextstrain clades from which they derive, as reported in column “parent” in the same table. A subsequent run of the official pangolin tool, executed a few months later, better interpreted S2 as line C.18 and S10 as B.1.177.33, reported as a mostly Italian lineage [28]. Both lineages were not annotated in the GISAID data, as they were recognized and added to the Pango collection at a later date. 

### 2.3. Mutational Characterization of Growing Subsets

The subsets were further characterized by determining their mutational content. For each identified subset, the virus sequences were aligned to determine their consensus sequence. The consensuses were then aligned between them and with the consensus of the major clades in a multiple alignment, where only positions which changed with respect to a reference sequence (Wuhan-Hu-1, Refseq NC_045512.2) [29], i.e., either the original or the mutated base, are reported (Table S2).

In Table 2, all the mutations observed in each subset are reported, organized by subgenomic mRNA/ORF. For ORF1ab, they are also organized by encoded peptide. The number of sequence variations ranges from 11 (S1) to 32 (S4) and, as expected, tends to be higher in subsets which appeared more recently. While some variations are ‘private’ for a specific subset, others are shared among multiple subsets deriving from the same clade, marked as ‘C’ in the ‘subsets’ columns. Among the “clade” variations, some (5′UTR:C241T, ORF1ab:C3037T, RdRp:P323L, S:D614G) are present in all subsets, as they all derive, directly or indirectly, from 20A. Subsets S2–5, i.e., all sub-branches of clade 20B, in addition, carry the variations N:R203K and N:G204K. S2 and S3, belonging to the same family 2–3, also have substitutions which define their parent clade 20D: nsp3:T428I and 3C-like proteinase: G15S. S8–10 are subgroups of clade 20E (EU1), from which they inherit the missense variations S:A222V, N:A220V, and ORF10:V30L, as well as a number of synonymous variations in ORF1ab and M. 

“Branching” mutations, reported as ‘B’ or ‘b’ in Table 2, depending on whether their frequency within the subset is higher or lower than 80%, are associated with branching from the original clade and might potentially provide novel features to the carrying viruses. Some of them are interesting as they characterize one or more subsets and may be involved in determining their expanding behavior, as a result of positive selection.

S1, corresponding to Pango lineage B.1.258, shows the characteristic S:N439K protein change found in two distinct clusters spreading in 2020 in Europe, after starting in Scotland and Romania [30]. It also includes two missense substitutions in the nsp3 (I1683T) and helicase (H290Y) non-structural protein genes.

The subset family, including S2 and S3, has several sequence changes, but the sole subset-specific non-synonymous mutation is the S166A substitution in 2’-o-ribose methyltransferase, which characterizes practically all (97.8%) genomes in the S2 subset and which mostly includes isolates from Campania, in Italy. This mutation appears at lower frequency (53.9%) in S3 only because it includes S2.

S4 corresponds with VOC 20I/501Y.V1 and contains, as expected, all mutations already described for this variant [31], including multiple changes in the spike protein, such as N501Y, P681H, H69-/V70-, and W144-, as well as other mutations such as ORF8:Q27, which cause the truncation of its protein products, D3L and S235F, in the nucleocapsid protein and the combined deletion of amino acids 3675–3677 in Nsp6. In addition to this list of variant defining mutations, we observed a mutation in helicase (K460R), present in about 51% of S4 genomes, clustered in a well-defined branch, not identified by itself as an independent subset only because it is smaller than the set threshold. The mutation is not limited to Italy and was probably imported a short time after the first appearance of the variant. 

S5 corresponds with Pango lineage B.1.1.39 and is mainly characterized by two amino acid changes in the nucleocapsid protein N: V72I and P199S, but also includes a number of synonymous mutations in ORF1ab and ORF6.

S6 and S7 constitute a family where S7 corresponds with clade 20A.EU2, and S6 represents about 20% of it. Furthermore, both are characterized by nine missense mutations located in proteins, including nsp4, RdRp, helicase, spike, ORF3A, and N, as well as several other synonymous ones. 

The three subsets S8, S9, and S10 are unrelated groups, all deriving from clade 20E (EU1) and differing for specific missense mutations as well as a few synonymous changes. S8 includes missense mutations nsp6:A54S, present in all members, and S:Q675H and ORF8:P30L, clustered in a subtree of slightly over 50% of the sequences not identified as a subset. S10, coincident with Pango B.1.177.33, is similarly characterized by missense mutations nsp3:T1456I, S:G639S, and N:P365S. S9 is essentially identical to the parental 20E (EU1), except for a single synonymous mutation (ORF8:C27944T), which is the only element which could justify the branching from the parental clade. It is noteworthy that this subset, although less defined than the other two, has also been detected in subsequent months (see below under “Tracing of variants”).

### 2.4. Structural Analysis

**Figure 3:**
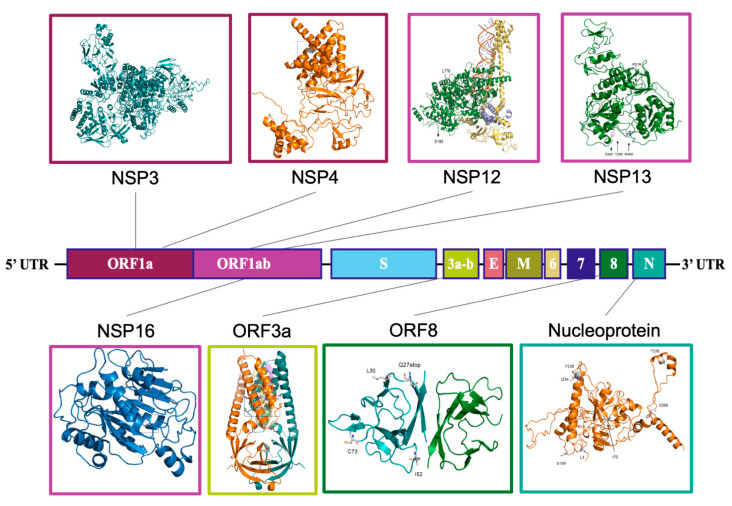
Genome layout of genes/proteins carrying mutations emerged during the year 2020. The approximate positions on the SARS-CoV-2 genome of genes coding for proteins carrying mutations from the year 2020 were subjected to structural analysis. The protein structures are displayed as ribbon models and enclosed in frames colored as the corresponding ORFs.

The mutations characterizing each subset were further analyzed, with an attempt to predict their effect on the 3D structures of the affected proteins by means of in silico mutagenesis and/or homology modeling, as appropriate. The analysis of the effect of the amino acid changes to the protein was mostly limited to ‘branch’ mutations, possibly directly related to some propagation advantage, and only in some cases including clade-related ones. The results are reported in the synthesis in Figure 3 and Table 3 and in detail in Figures S1–S8 of the SI document. Table 3 also provides a list of all analyzed mutations and their main expected effects on protein structure.

Focus is given to mutations in proteins other than spike, as this protein, along with its mutants, have already been extensively discussed in the literature. Among mutations which characterize S1, spike change N439K has already been shown by others to increase ACE2 binding [30,32] and possibly confer resistance to antibodies [33,34]. In addition, H290Y substitution in helicase (Nsp13) was found to generate an additional H-bond to the peptide bond of E261 (Figure S1, inset H290Y) and may promote stacking of the aromatic ring with F262, resulting in the stabilization of the protein with a predicted ∆∆G of +2.790 kcal/mol. Finally, another S1 mutation involves I1683 in Nsp3, an ORF1a peptide, and its change into T is predicted to reduce hydrophobic interactions with residues V1678 and L1685 (Figure S2, inset I1683T).

In S2–3, the S166A change in the 2′-O-ribose methyltransferase enzyme (Nsp16) causes a loss of a hydrogen bond to N210 and produces hydrophobic interactions to L59 and L126 at the same time (Figure S3). These changes lead to an overall predicted protein stabilization (+0.425 kcal/mol). 

S4 includes several mutations, most of which, especially those occurring in spike protein, have already studied elsewhere as characterizing VOC 20I/501Y.V1. In addition, three distinct amino acid changes involve the previously mentioned Nsp3 peptide, in positions apparently not involved in specific interactions, with the possible exception of T183I, which removes an OH group and potentially abolishes polar interaction to Q185 or Q180 (Figure S2, inset T183I). Another mutation, found in a sub-branch of S4, involves residue K460 in helicase. Its change into R promotes new interactions with Y457 via a hydrogen bond and D458 by electrostatic interaction, resulting in an expected increase in stability (+1.254 kcal/mol) (Figure S1, inset K460R).

S5 is characterized by two changes in N: V72I, which was predicted to increase hydrophobic interactions with neighboring residues, and P199S, which can interact with S197 through a hydrogen bond (Figure S4, inset P199S). 

S6–7, corresponding to 20A.EU2, are characterized by nine missense mutations located in proteins, including nsp4, RdRp, helicase, spike, ORF3A, and N, among others. S477N, in the receptor binding domain of the spike protein, was not analyzed in detail, because it has already been investigated by others, who reported an increased ACE2 binding and increased resistance to multiple antibodies [32,35,36]. Of the several changes involving ORF1ab, one in Nsp4 (M324I) affects the hydrophobic interactions to L321 and L323 of the opposite helix (Figure S5), and two in RdRp (Nsp12) include A185S, which can cause the formation a H-bond to V182/N213, and V776L, which can probably increase hydrophobic interactions to H752, F753, and Y748 (Figure S6). Of the changes in helicase Nsp13, still in ORF1ab, E261D may introduce a H-bond to S259 and abolish H-bonds to Y324 and H290 (Figure S1, inset E261D). Finally the substitution of a glutamine to histidine in position 57 is predicted to increase the stability of the ORF3a protein, with a ∆∆G of +1.620 kcal/mol (Figure S7), while the change A376T in N may potentially introduce a H-bond to K374 (Figure S4, inset M234I/A376T).

The most interesting evidence in S8 is a putative stabilization of ORF8 product (+1.620 kcal/mol), resulting from the substitution of Proline 30 in Leucine (Figure S8, inset P30L).

S10 is characterized by three ‘branching’ amino acid changes, in ORF1ab Nsp3, S, and N. In Nsp3, T1456I produces an exposed isoleucine which is predicted to remove polar interactions to N1457 in a loop at the C-terminal of a α-helix (Figure S2, inset T1456I), while in N, P365S, also exposed in a loop, appears to increase local flexibility (Figure S4, inset A220V/P365S).

### 2.5. Tracing of Variants

**Figure 4:**
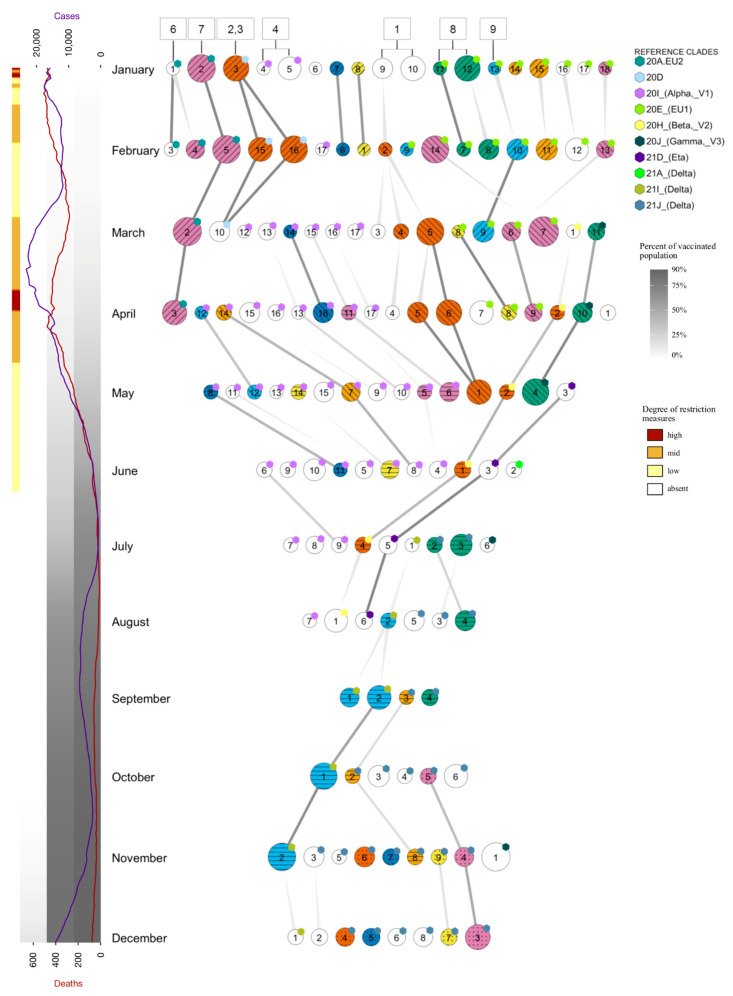
Tracing of growing subsets over the year 2021. Each horizontal line corresponds to a dataset consisting of sequences available at the beginning of the month indicated on the left side. The circles correspond to the subsets identified with the procedure used for Figure 2 and Table 1, with the area proportional to the number of sequences, and colors and patterns are used to highlight the subsets which share the same consensus sequence. The connectors highlight relations between subsets taken from subsequent months, based on the sequences present in both datasets and shared by the two subsets, as described in Methods, with the gray intensity proportional to the number of sequences shared by them. A symmetric relationship is indicated by connectors of constant thickness, meaning that most (>80%) sequences from each subset are contained in the other one and vice versa. In contrast, lines with progressively reduced thickness indicate that only sequences of one subset are mostly (>80%) contained in the target one, but the opposite is not true. The colored sticker on the top right corner of each subset indicates the clade to which the subset corresponds to or derives from, as indicated in the legend. Subsets in the ‘January’ dataset are labeled with a number indicating the corresponding subset in the ‘first year’ dataset of Figure 2. On the left, the number of positive cases and deaths are reported in time. The gray gradients in the background indicate the percent of the population vaccinated with a first, second, and third dose, respectively, on the right, center, and left side. Red, orange, and yellow colors are used to indicate the prevalent level of restrictions imposed by the Italian government (from most to the least restrictive), as defined in the Italian Decree (G.U. n° 275 4 November 2020). The reference graph on the left was produced by using cases/deaths and vaccination numbers published by “Civil Protection Department” [47] and by “Ministry of Health” [48], respectively.

The emergence and propagation of SARS-CoV-2 variants in the last months of 2020 stimulated the development of a tracing system, constructed by using the previously described subtree-searching tool and directed to the identification of novel emerging subtrees. The tracing system was used to extract and analyze a new sample, from the collection of viral sequences available in public databases, at the beginning of each month. Considering that, after the remarkable reduction experienced in summer 2020, the pattern of virus spread in Italy has substantially changed, and the sampling criteria were modified for the tracing procedure by shifting the focus previously given to Northern Italy and neighboring countries to the whole Italian territory. The results of the analysis, carried out during the months between January and December 2021, are reported in Figure 4, where each horizontal line corresponds to the subsets (circles) identified by analyzing the data available at the beginning of the month indicated on the left side, collected as described under Materials and Method, with the sole exception of the ‘January’ sample, collected on 15 February, rather than 15 January, to compensate for the delayed submission occurred, mainly in Italy, in the months around the end of 2020. The connectors highlight relationships between subsets identified from subsequent datasets.

The S1–10 subsets, identified by analyzing the previously described 2020 dataset, were compared (see Materials and Methods and Figure S9) to the subsets of the January dataset, corresponding to an equivalent time window (year 2020), but with a shifted focus centered on the whole Italian territory. The 2020 subsets with a counterpart in the ‘January’ subsets are indicated in Figure 4 by boxes connected to the circles of the January row. In particular, we observe that, out of ten 2020 subsets, eight correspond to one or more subsets of the ‘January’ dataset. Specifically, S1 coincides with the subset family formed by January subsets 9 and 10, although subset 9 also includes an additional mutation in the N protein, S194L, which is present in a subtree containing just over 50% of the subset sequences, which corresponded with Pango line B.1.258.14, mainly circulating in Italy [37]. The family composed by S2 and S3 corresponds to the single subset 3, while the other 2020 family, composed by the S6 and S7 subsets, found a counterpart in subsets 1 and 2, respectively, with subset 1 showing an additional mutation in ORF3a. S4 was mapped in the new dataset to the family composed by subsets 4 and 5, both containing virus genomes belonging to VOC 20I/501Y.V1, but further characterized by two additional sequence changes in ORF1ab, T1567A, and Q3966R, present in over 50% of its sequences, which were absent in the 2020 S4 subset. Regarding the S8, S9, and S10 subsets, derived from clade 20E EU1, the first expands into the subsets 11–12, while S9 overlaps with subset 13. S10, in contrast, does not have a corresponding subset in the January dataset, but its sequences are present within a larger subtree, which is too big to be selected as a novel expanding subtree. S5, which includes only Swiss samples, does not find a counterpart in the ‘January’ dataset focused on sequences from Italy, as expected.

The graph reported in Figure 3 demonstrates the propagation of each subset in the following months until they disappear, either having been superseded by other virus variants, or having become established branches which exceed the maximum size set for an expanding subset. In this last case, new subsets are often generated as novel, and positively selected mutations appear within the branch. Examples of these chains are the January subset 2, corresponding to the original family S6–7, which may be followed until April, until it disappears in agreement with a strong reduction in the corresponding 20A.EU2 variant, as also observed in other reports after March [38]. Similarly, subset 3 (S2–3 in 2020), only continues up to March subset 10, following the destiny of the parent clade 20D, which also shows a reduction after the first months of 2021 [39]. The January subsets 4 and 5 do not continue in the following months, as this lineage, corresponding to variant 20I/501Y.V1, became shortly predominant and is not therefore reported any more as a subset; however, starting from March, the procedure highlighted new growing subsets deriving from the same lineage, which represent potentially interesting sub-variants. They may be easily recognized in the figure by the corresponding tag label, such as in the case of subset 15 from the March dataset, which may be followed up to June, and subset 14 from April, which continues until June. The path starting from subset 15 diverges from the original lineage for the presence of two mutations in ORF1ab (G1125C and T4265I) and one in N (L139F). Conversely, the path starting from subset 14 is characterized by two mutations in ORF1ab, P4619L and P6376S, which are not reported in any of the lineages derived from B.1.1.7 (the original Alpha variant), supporting the hypothesis of potentially new unreported sub-variants. Subsets 9 and 10 from the ‘January’ dataset (S1 in 2020) are partially related to subset 2 of the February dataset, and continue in subsequent months through May, including multiple subsets with each month. As reported in Table S3, the consensus of these subsets contains the signatures of the Pango lineage B.1.258, but also includes additional changes in ORF1ab (NSP9:M101I, RdRp:V720I, and Helicase:A598S) and S (69/70del), also described for several B.1.258 sub-lineages [40], including B.1.258.7, B.1.258.17, B.1.258.20, and B.1.258.24, observed as small subtrees which derive from the main lineages corresponding to the above subsets, but too small to be selected as separate subsets. The January subsets, 11–12 and 13 (S8–9), corresponding to two distinct subgroups of clade 20E (EU1), both propagate in the next few months, up to February and March, respectively. In the January dataset, there are actually other subsets (from 14 to 18) also belonging to clade 20E (EU1)—most of them do not last for long, except for subset 18, observed until April. Some derive from known B.1.177 sub-strains, specifically B.1.177.75 (subsets 14 and 15) and B.1.177.83 (subset 16), both reported in Pango as primarily Italian lineages [41,42].

The graph also highlights additional paths unrelated to the subsets identified in the first year. A long-lasting one starts in March with subset 1 and ends up in August; it derives from clade 20H (Beta, V2), one of the variants of concern, reported at the end of 2020 in South Africa [43], and appeared with few cases in February in Italy, where it persisted over the following months without expanding very much. A second lineage, seen from March (subset 11) to May (subset 4), corresponds to another variant of concern, 20J (Gamma, V3), which originated in Brazil at beginning of 2021 [44]. A third lineage, starting in May with subset 3 and continuing until August, corresponds to variant 21D (Eta). Note that the first samples of this variant appear in Italy already in February, but they reach the features of a growing subset in April. The second half of 2021 was dominated by VOC 21A (Delta) which, after its first appearance in India in late 2020, subsequently spread around the world [45,46]. In Italy, this variant emerged in May and became predominant in the following months, including most of the identified subsets. Some subsets, after their first appearance, are conserved in the following months, forming chains of interconnected subsets, as in the case of the lineage starting in August with subset 2 which remained active until December with subset 1, retaining the features of the original Delta variant (Pango B.1.617.2) (Table S3), or the lineage beginning in July (subsets 2–3) and continuing until September, which tends to include genomes close to Pango AY.4 and AY.9 during its propagation, but without reaching the size required to be selected as separate subsets. Two additional lineages, starting in September with subset 3 and in October with subset 5, respectively, have been found to correspond with AY.122 and AY.43, respectively, i.e., two “Delta” sub-lineages which spread mainly in Europe. 

The emergence and propagation of growing subsets was put in the context generated by disease progression and containment measures by plotting, alongside the subsets, the daily number of infections and deaths officially ascribed to COVID-19, as well as the fraction of Italian population vaccinated with one, two, or three doses, represented as three background stripes whose gray level ranges between white (0%) and black (100%). Restriction measures are also indicated as yellow, orange, or red, according to the corresponding government-imposed limitations, as indicated in Figure 4. As arguably expected, the number of growing subsets varies during the year and is highest in the early months. When virus propagation was high, restrictions were limited and vaccination degree was null or just starting. After the restrictions imposed between March and May, the progressive decrease in the number of cases is accompanied by a concomitant reduction in the number of growing subsets, due to both the interruption of most subset chains characterizing the earlier months and the strongly reduced appearance of novel subsets. It is likely that the increasing number of second doses (20%→40% between May and July), as well as higher temperatures and better overall climatic conditions, played a role. However, during these months, virus propagation and adaptation was by no means over, resulting in the appearance of new lineages (Delta and its sub-variants) starting from July/August, which will increase in the following months and slowly substitute the previous variants (20E (EU1) and 20I (Alpha)). The new variants contributed to the increasing numbers of virus infections in the last few months of the year; however, in this phase, the epidemic was characterized by a much lower mortality rate compared to March 2022, which has persisted up to now.

## 3. Discussion

Tracking SARS-CoV-2 lineages is a public health priority, important to identify expanding virus variants and to rapidly detect sequence changes that may influence the infectivity, severity, or immune susceptibility of SARS-CoV-2. The procedure described in this study can rapidly identify expanding clusters in large phylogenetic trees containing thousands of nodes and has been used to identify expanding clusters potentially corresponding to emerging virus variants. Although the selection of growing subtrees mainly relies on the date of sample collection to identify subsets, these may be automatically annotated by taking advantage of other epidemiological traits attached to the selected virus sequences. Unlike other procedures for phylogeny partitioning, where clusters are often defined based on low within-cluster genetic distances [49,50], in this case, the selection criteria were based on a simple but effective method that does not depend on the knowledge of the specific amino acid changes that produce tree clusters, but looks at the size and at the apparent expansion rate of each subtree, assessed as the skewness of the distribution of collection dates of viruses descending from the subtree’s most recent ancestor (MRA).

Analysis of a dataset consisting of viral sequences from Italy and neighboring countries correctly identified all known virus variants circulating in this area during 2020 and highlighted a number of different scenarios. For example, one of the identified subsets (S4), found to correspond with the so-called “UK” variant, was already identified as early as in December, in agreement with other reports [15]. The subset, although fairly small, was characterized by a relatively high skew value, indicative of rapid expansion well before the explosion which characterized its spread in Italy during the first few months of 2021. This behavior shows the ability of the procedure to highlight the potential of successful virus strains at an early stage. In a different scenario, the S7 subset was found to correspond to the strain 20A.EU2 (B.1.160) which, unlike the previous one, was effectively circulating around Italy since September [16,51], was already well established by the end of 2020, and was about to undergo various steady-state and reduction phases in early 2021. This subset scored well, although characterized by a lower expansion rate at the end of the observation period, mainly because of the higher number of virus samples. The third main virus variant circulating in 2020 was 20E (EU1) (B.1.177), which represented the majority of the circulating viruses in the analyzed area in the second half of the year [15,16,51]. In this case, the procedure, as expected, could not select the entire clade, whose size was well above the defined 5% thresholds, but identified three smaller subtrees, potentially corresponding, at least in part, to new lineages originating from speciation of the parental clade. Subsequent analyses confirmed this hypothesis, as the S10 subset at the time of analysis, belonging to the main Pango B.1.177 lineage, could only be recognized as B.1.177.33 a few months later, with a subsequent run of the pangolin tool [52]. This was not an isolated case, as also S2 was similarly assigned to Pango lineage C18 at a later date. This, therefore, shows that the tool can be effective at identifying new strains in the early stages of their emergence. 

Further analysis of the subset consensus sequence was directed to identify and characterize mutated sites. The differentiation between ‘clade’ and ‘branch’ mutations highlights the changes associated with the branching from the original clade, potentially involved in determining the expanding behavior of the subtree, and possibly providing novel features to the viruses carrying them. In some cases, the identified mutations are found in multiple subtrees, apparently arising independently, potentially as a result of adaptive evolution, as in the case of the H69-/V70- deletion, in the terminal loop of a helix loop in the S1 domain of the spike protein. This deletion has been frequently found to co-occur with other mutations in the spike receptor-binding domain, such as N439K, Y453F, and N501Y [40,53,54], thus suggesting that it may increase overall fitness in concert with mutations that would otherwise be neutral.

Structural analysis of protein sequences carrying one or more of the mutations identified in the 2020 dataset provided additional insight into the possible consequences of such changes on protein conformation and/or stability. The analysis demonstrated that several amino acid changes can alter the normal interaction with neighboring sites and, in some cases, also act on the conformation stability of key SARS-CoV-2 proteins. Among the affected viral proteins, the nsp12 catalytic subunit is part, in combination with nsp7 and nsp8, of the more complex machinery corresponding to RNA-dependent polymerase (RdRp), that is fundamental to replication and transcription and plays an additional role in assessing transcriptional fidelity, via its proofreading activity [55]. RdRp is also known to coordinate the discontinuous transcription process described in the introduction, operating according to the prevailing “leader-to-body” fusion model [4]. More recently, the nucleocapsid (N) protein [7,56], encoded by sgN transcript, has also been found to play a role in regulating the discontinuous transcription process through its C-terminal domain that retains the interaction and consequential regulation of transcription with TRSs sequences [57]. As both RdRp (nsp12) and the nucleocapsid (N) proteins are key components of the viral replication and transcription machinery, it is conceivable that emerging mutations in RdRp (including those found at position 14,408) [58] and N proteins could modify their transcription and proofreading activities, hence altering the mutation rates of SARS-CoV-2 for survival and adaptation during the evolution of the pandemic.

The ability of the tracing system to monitor the propagation of emerging subtrees over time provides an opportunity for characterizing the evolution of the virus pandemic, highlighting lineages which are conserved and possibly expanded in multiple subsets. The system is based on a combination of two procedures, which involve a setup for pairing subsets identified in distinct runs of the subtree-searching tool, even if executed on partially overlapping or even completely different sampled datasets. In the first, similarly to other cluster matching tools [49], subsets are paired on the basis of their degree of overlap, using the percentage of common elements between two datasets as the main parameter. The procedure is typically used to pair subsets sharing a small fraction of samples, as those obtained by repeated random sampling of the same population. It is fast and effective, and can also be used to pair subsets selected according to other criteria, such as epidemiological or clinical data, including the risk group or stage of infection. The second procedure, set up to compare subsets by using their consensus sequence, complements the previous one and may even be used to compare non-overlapping datasets, such as those sampled from different geographic areas. In its present form, the tracing system pairs subsets by using a combination of techniques, and can distinguish fully corresponding subsets from subsets including another one or extracted from another. The sequence-based pairing reinforces the element-based pairing and, in some cases, complements it by highlighting pairs not detected with the first method. Taken together, they help to pair very heterogeneous datasets, as similarities can easily be detected between subsets sharing a very limited number of viral sequences or even none, as long as they share the same mutations.

The tracing system helps to easily follow the fate of previously identified virus subsets as well as to detect new emerging lineages. Tracing of the main strains that appeared during 2020 shows that clade 20A.EU2 persisted in the subsequent months as a single homogeneous lineage, before fading out at the beginning of May 2021, while clade 20E (EU1) had already differentiated into distinct subsets in January, and two additional lineages had been established alongside the original variant. Subsequent analysis helped to demonstrate their correspondence with Pango B.1.177, matching Pangos B.1.177.75 and B.1.177.83, respectively. The tracing system was effective in showing the strong expansion of the 20I (Alpha v1) variant at the beginning of 2021, with a peak between March and June, when the original variant split into several sub-sets introducing just a few changes in ORF1ab, and its later reduction concomitant with the spread of the Delta variant. During spring and summer, the strong reduction in the number of infected cases is reflected in the contraction of the number of subsets, which probably contributed to the definitive disappearance of the 20I (Alpha) variant, which was completely replaced by the Delta variant from August onwards, i.e., virtually the only variant circulating in Italy up to the end of 2021. Looking instead at the spread of the Delta variant in Italy, the tracing system again appears to be effective in the early identification of successful virus strains, as Delta-type subsets are detected in samples collected in May, i.e., at the first possible opportunity. In fact, although individual Delta sequences in Italy date back to March, the spread of the variant was only recognizable in May and June [59]. The Delta subsets identified in subsequent samples until September only show minimal changes compared from the original variant, and are all classified as Pango lineage B.1.617.2. Starting from September, a number of sub-variants were recognized, and later related to Pango lineages AY.4, AY.43, and AY.122. 

Overall, the tracing system can follow the diffusion, persistence, and disappearance of novel variants and is effective in the early identification of new virus strains if they spread fast in the observed population. Relating the dynamics of growing subsets emergence to clinical evolution of the epidemic, it is clear that a higher number of cases are strongly associated with a more frequent appearance of novel subsets. Although not all of them result in subsequent well-defined lineages, many of them do, and the process is certainly more evident when the number of cases is high or increasing, allowing the virus to get fitter. Overall, the presented data support the idea that vaccination, together with mobility restrictions, barrier devices, and interpersonal distancing, can be very effective at reducing virus propagation and new variant emergence. Although the efforts presented here are proven to identify emerging SARS-CoV-2 clusters, it remains strongly dependent on the submission of timely data to public databases. Moreover, of course, it is the responsibility of researchers, clinicians, and public health authorities to combine this with other tools, to correctly analyze the available information, and to choose the right strategies to effectively fight the SARS-CoV-2 pandemic.

## 4. Materials and Methods

### 4.1. Sequence Datasets and Phylodynamic Analysis

Sequences and metadata were downloaded from GISAID on 8 January 2021 for the analysis of the first year (n = 330,132) and on 16 December 2021 for tracing (n = 6,079,190). Phylodynamic analysis was based on the *Nextstrain/ncov* workflow, optimized for SARS-CoV-2 analysis [17,60]. *nextstrain.cli* version 2.0.0.post1 was used to analyze 2020 data, while *nexstrain.cli 3.0.3* was used for the datasets included in the tracing analysis. Within the *ncov* workflow, sequences from the starting dataset are filtered by removing genomes based on general quality criteria and custom exclusion lists, and aligned to a reference sequence (typically Wuhan-Hu-1, Refseq NC_045512.2). The aligned sequences were then sampled according to a set of rules based on a focus and a context, and fed to IQ-Tree [61] to infer phylogeny. After a final refinement and time-scaling with *TreeTime* [17,62], the JSON output was used for visualization in Auspice [63].

In the present work, the predefined set of filtering criteria was used, which excludes sequences shorter than 2700 nts, with an ambiguous sample collection date, or included in the default Nextrain exclusion list (where the Nexstrain team annotates samples with known issues, such as annotation mistakes, duplications, sequencing errors or too much divergence from the reference virus sequence. The exclusion list is available online [64]. Specific sets of sampling rules were defined; the rules used for the 2020 dataset and all 2021 datasets are reported in *builds_2020.yaml* and *builds_2021.yaml*, respectively (in the Supplementary Materials). As also reported in the ‘Results’ section, the focus sequences included, for the 2020 build, all the available Italian ones (mostly from northern Italy), and a comparable number of sequences from neighboring countries, taken in proportion to population size and SARS-CoV-2 infection incidence, as derived from the ‘European Centre for Disease Prevention and Control’ reports [65]. Sequences were randomly selected when the number of available samples exceeded the desired target number. The analyzed dataset was completed by a random sample from the rest of Europe and from non-European countries, used as context, until reaching a maximum of additional 1000 sequences. For the 2021 monthly datasets, only sequences collected up to the 1st and available on the 15th of each month were used. The focus consisted of a random sample of 3000 sequences of the last 12 months from all of Italy, plus a smaller sample from the previous months; the rules for the context were set for 2020. A list with GISAID accession numbers and appropriate acknowledgement to sequencing laboratories is provided in Table S4.

### 4.2. Identification of Growing Virus Subsets

Growing virus subsets were detected by processing the phylogenetic tree generated by the *ncov* workflow with a custom-developed method, implemented as a PHP script. The procedure assigns a score to each internal node of the tree on the basis of the skewness of the collection date distribution of its children leaves, corrected according to the number of leaves. Subtrees with a score of at least 5, with a size between 30 and 5% of the total number of leaves, and including leaves sampled in last 20 days of the analyzed period are selected as growing subsets.

The score is calculated as follows:

*Score* = (*w*1 × (−*s*)) + (*w*2 × (1 + log10 (*n/N*)), (1)

where *s* is the skewness coefficient; *n* is the number of the leaves in the subset; *N* is the total number of leaves; and *w*1 (default 20) and *w*2 (default 7) are two weights used to modulate the effect of skewness and the subset size, respectively. The skewness is based on the Fisher–Pearson coefficient of skewness and is calculated with the following formula:

(2) $$\frac{\sqrt{N\left(N-1\right)}}{N-2}\frac{\sum_{i=1}^{N}{\left(Y_{i}-\bar{Y}\right)}^{3}/N}{S^{3}}$$

where *N* is the sample size, *Ȳ* is the mean value, and *S* is the standard deviation.

The original tree is, finally, modified by adding relevant data on the selected subtrees, such as the score and the skewness, and is visualized in Auspice. The procedure generates a report, in the form of a table, where a number of details are indicated for each subset, including the inferred date of first occurrence, the subset size, and the geographic distribution.

### 4.3. Detection of Mutated Sites

The sequences from each subset were aligned using *mafft v7.475* [66,67] or *nextaling v1.2.0* [68] to generate a consensus. Then, a multiple alignment with the consensus of each subset and of the major clades was generated, where either the original or the mutated base were reported for each position which changed with respect to the reference sequence (Wuhan-Hu-1, Refseq NC_045512.2). For substitutions occurring in translated sequences, the amino acid change was also identified and reported. All the steps of this procedure were encoded in a tool, written in PHP.

### 4.4. Impact of Mutations on Protein Conformation and Stability

Available SARS-CoV-2 protein structures were retrieved from the Protein Data Bank [69]. Proteins without a complete structure were taken from the *I-TASSER* [70] repository containing a collection of protein structures which were predicted using an ab initio fragment-based approach. Molecule visualization relied on *PyMOL* [71] or *CHIMERA* [72]. In silico mutagenesis was carried out using the ad hoc tools available within the *PyMOL* or *CHIMERA* function set. The impact of mutations on the protein structure stability was predicted with the server DynaMut [73]. The server implemented a method based on the normal mode analysis of protein dynamics trajectories. DynaMut was applied only to experimentally solved structures. 

### 4.5. Subset Relationships with Nextstrain Clades/Pango Lineages

Subsets were initially assigned to a Nextstrain clade and/or a Pango lineage, using the information annotated for each leaf in the tree file. The subset was considered ‘coincident’ with a given clade/lineage, if they shared at least 95% of their elements, and ‘derived’ from it, if all subset members are contained in the clade/lineage but constitute less than 95% of its members. At a later stage, the relationship of each subset with Pango lineages was reassessed by using the subset consensus sequence as an input, as well as the official pangolin tool [52] (version 3.1.14) and the pangoLEARN algorithm with default parameters.

### 4.6. Subset Tracing

The procedure developed to follow the destiny of the subsets in the following months is based on a tool which highlights the relationship between subsets from different datasets, determined using the percentage of common leaves as a main parameter. For each comparison, in which a test dataset is checked against a reference dataset, the tool uses the output generated during the process of subset identification and finds the relationships between subsets, connecting them on the basis of their degree of overlap. Given the two paired subsets from different datasets, two ratios are calculated by dividing the number of leaves shared by the two subsets by the number of leaves shared by each subset with the dataset from which the paired subset derives. Based on the product of two ratios, the following kinds of relationship may occur:

(a) full correspondence, if the product is 0.8 or more;
(b) partial correspondence, if the product is less than 0.8, but at least one of the two ratios is more than 0.8;
(c) no relationship, in the other cases.

Additional relationships between subsets from different datasets may also be obtained by matching them on the basis of their consensus sequence, with subsets considered identical when sharing the same consensus.

Membership of a subset in a Nextstrain clade is assigned on the basis of the presence in their consensus sequence of the clade signatures used by the *ncov* workflow version 3.0.3, reported in *clade_signatures.txt* in the Supplementary Materials.

## 5. Conclusions

A procedure which can identify expanding branches in large phylogenetic trees was set up and used to identify growing subtrees potentially corresponding to emerging viral variants. Analysis of viral samples collected in Italy and neighboring countries during 2020 correctly identified all known viral variants circulating in this area. Structural analysis of proteins from this dataset, carrying sequences changes, provided insight to the possible consequences of such changes on protein conformation and/or stability. 

A tracking system, set up to monitor the propagation of emerging variants, provides an opportunity to follow virus evolution by highlighting new lineages and their further expansion into multiple subsets, while detecting sequence changes that may influence SARS-CoV-2 infectivity, severity, or immune susceptibility. Overall, the tracking system appears effective in following the spread, persistence, and disappearance of new variants, and can facilitate the early identification of new viral strains which spread rapidly in the observed population.

## Figures and Tables

**Table 1:** Annotated list of growing subsets identified in the 2020 dataset.

Family	Subset	Date	Internal Subsets	# Viral Seqs	Score	Skew	Origin		# Sources		Sex			Clade		Related Pango Lineage	
							Italy	Others	Div.	Cent.	F	M	U	Parent	Is	Name	Rel.
	1	4/2020		61	11.32	−0.84	41 (67%)	20 (33%)	6	10	18	31	12	20A		B.1.258	ovr
**2−3**	2	6/2020		47	7.21	−0.68	47 (100%)	0 (0%)	3	5	23	24	0	20D		C.18	not
	3	4/2020	2	91	14.33	−0.93	56 (62%)	35 (38%)	8	12	34	34	23	20D		B.1.1.1	par
	4	10/2020		43	54.79	−3.07	25 (58%)	18 (42%)	6	12	18	16	9	20B	20I/501Y.V1	B.1.1.7	ovr
	5	3/2020		45	6.65	−0.66	4 (9%)	41 (91%)	3	3	7	6	32	20B		B.1.1.39	ovr
**6−7**	6	4/2020		31	7.77	−0.77	20 (65%)	11 (35%)	4	7	6	17	8	20A.EU2		B.1.160	par
	7	6/2020	6	164	8.66	−0.56	68 (41%)	96 (59%)	6	12	29	56	79	20A	20A.EU2	B.1.160	ovr
	8	6/2020		52	20.53	−1.33	27 (52%)	25 (48%)	5	8	19	13	20	20E (EU1)		B.1.177	par
	9	7/2020		33	5.81	−0.66	22 (67%)	11 (33%)	5	6	9	16	8	20E (EU1)		B.1.177	par
	10	8/2020		49	13.77	−1	47 (96%)	2 (4%)	4	5	13	34	2	20E (EU1)		B.1.177.33	not

The ‘family’ column indicates the overlapping subsets. ‘Date’ is the inferred date of the subset earliest appearance. ‘Score’ is the parameter used to identify emerging subsets, as detailed under ‘Methods’. ‘Origin’ indicates the number and percent of samples collected in Italy and in other countries. ‘Source’ reports the number of administrative divisions (Div.) and laboratories (Labs) from which the sequences are sourced. ‘Clade’ reports the parent as well as, when available, the corresponding Nextstrain clade. ‘Pango’ contains the closest Pango lineage and its relationship with the subset, annotated by using the following schema: ovr—Pango lineage overlaps with the subset; par—Pango lineage corresponds with the Nextstrain parental clade from which the subset derives; and not—Pango lineage not initially recognized as it was not available in the Pango collection at the time of analysis.

**Table 2:** List of mutated sites observed in the subsets, sorted by genomic coordinates.

Variant	Variant (ORF1ab Peptide)	Nucleotide	Subset									
			1	2	3	4	5	6	7	8	9	10
**5UTR: C241T**	-	241	C	C	C	C	C	C	C	C	C	C
**ORF1ab: V60V**	leader: V60V	445								C	C	C
**ORF1ab: S216S**	nsp2: S36S	913				B						
**ORF1ab: F924F**	nsp3: F106F	3037	C	C	C	C	C	C	C	C	C	C
**ORF1ab: T1001I**	nsp3: T183I	3267				B						
**ORF1ab: T1246I**	nsp3: T428I	4002		C	C							
**ORF1ab: T1426T**	nsp3: T608T	4543						C	B			
**ORF1ab: Y1635Y**	nsp3: Y817Y	5170								B		
**ORF1ab: A1708D**	nsp3: A890D	5388				B						
**ORF1ab: T1788T**	nsp3: T970T	5629						C	B			
**ORF1ab: F1907F**	nsp3: F1089F	5986				B						
**ORF1ab: T2007T**	nsp3: T1189T	6286								C	C	C
**ORF1ab: I2230T**	nsp3: I1412T	6954				B						
**ORF1ab: T2274I**	nsp3: T1456I	7086										B
**ORF1ab: I2501T**	nsp3: I1683T	7767	B									
**ORF1ab: Y2594Y**	nsp3: Y1776Y	8047	B									
**ORF1ab: S2625S**	nsp3: S1807S	8140								b		
**ORF1ab: S2839S**	nsp4: S76S	8782										
**ORF1ab: V2955V**	nsp4: V192V	9130					B					
**ORF1ab: M3087I**	nsp4: M324I	9526						C	B			
**ORF1ab: G3278S**	3C-like proteinase: G15S	10,097		C	C							
**ORF1ab: F3329F**	3C-like proteinase: F66F	10,252		B	b							
**ORF1ab: A3623S**	nsp6: A54S	11,132								B		
**ORF1ab: S3675-**	nsp6: S106-	11,288–11,290				B						
**ORF1ab: G3676-**	nsp6: G107-	11,291–11,293				B						
**ORF1ab: F3677-**	nsp6: F108-	11,294–11,296				B						
**ORF1ab: Y3744Y**	nsp6: Y175Y	11,497						C	C			
**ORF1ab: D3897D**	nsp7: D38D	11,956		B	b							
**ORF1ab: Y4424Y**	RdRp: Y32Y	13,536		C	C							
**ORF1ab: A4577S**	RdRp: A185S	13,993						C	B			
**ORF1ab: P4715L**	RdRp: P323L	14,408	C	C	C	C	C	C	C	C	C	C
**ORF1ab: P4804P**	RdRp: P412P	14,676				B						
**ORF1ab: H5005H**	RdRp: H613H	15,279				B						
**ORF1ab: V5168L**	RdRp: V776L	15,766						C	B			
**ORF1ab: L5283L**	RdRp: L891L	16,111					b					
**ORF1ab: T5304T**	RdRp: T912T	16,176				B						
**ORF1ab: K5542R**	helicase: K218R	16,889						C	B			
**ORF1ab: E5585D**	helicase: E261D	17,019						C	B			
**ORF1ab: H5614Y**	helicase: H290Y	17,104	B									
**ORF1ab: K5784R**	helicase: K460R	17,615				B						
**ORF1ab: L6205L**	3′-to-5′ exon.: L280L	18,877						C	B			
**ORF1ab: L6668L**	endoRNAse: L216L	20,268	B									
**ORF1ab: K6958K**	2′-o-MT: K160K	21,138		B	b							
**ORF1ab: S6964A**	2′-o-MT: S166A	21,154		B	b							
**ORF1ab: A6997A**	2′-o-MT: A199A	21,255								C	C	C
**S: H69-**	-	21,767–21,769				B						
**S: V70-**	-	21,770				B						
**S: Y144-**	-	21,992–21,993				B						
**S: A222V**	-	22,227								C	C	C
**S: N439K**	-	22,879	B									
**S: S477N**	-	22,992						C	B			
**S: N501Y**	-	23,063				B						
**S: A570D**	-	23,271				B						
**S: D614G**	-	23,403	C	C	C	C	C	C	C	C	C	C
**S: G639S**	-	23,477										B
**S: Q675H**	-	23,587								b		
**S: P681H**	-	23,604				B						
**S: T716I**	-	23,709				B						
**S: T723T**	-	23,731		C	C							
**S: S982A**	-	24,506				B						
**S: D1118H**	-	24,914				B						
**ORF3a: Q57H**	-	25,563						C	B			
**ORF3a: L106L**	-	25,710						C	B			
**M: Y71Y**	-	26,735						C	B			
**M: L93L**	-	26,801								C	C	C
**M: I118I**	-	26,876						C	B			
**ORF6: L40L**	-	27,319					B					
**ORF7b: A15A**	-	27,800	B									
**ORF8: H17H**	-	27,944									B	
ORF8: Q27 *	-	27,972				B						
**ORF8: P30L**	-	27,982								b		
**ORF8: R52I**	-	28,048				B						
**ORF8: Y73C**	-	28,111				B						
**N: D3L**	-	28,280–28,282				B						
**N: V72I**	-	28,487					B					
**N: P199S**	-	28,868					B					
**N: R203K**	-	28,881–28,882		C	C	C	C					
**N: G204R**	-	28,883		C	C	C	C					
**N: A220V**	-	28,932								C	C	C
**N: M234I**	-	28,975						C	B			
**N: S235F**	-	28,977				B						
**N: P365S**	-	29,366										B
**N: A376T**	-	29,399						C	B			
**ORF10: V30L**	-	29,645								C	C	C
**3UTR: G32T**	-	29,706					b					
**3UTR: G60C**	-	29,734	B									

Columns 1 and 2 reports mutations detected by comparing the consensus sequence of each subset to Wuhan-Hu-1 (Refseq NC_045512.2), used as reference. Mutations are described following standard naming convention where the standard one-letter code indicates original and mutated amino acids, ‘*’ and ‘-’ indicate stop codons and deleted amino acids, respectively. Numbers indicate the amino acid position: in column 1 they are referred to the main protein encoded by each gene, while in column 2, the amino acid position is referred to the peptide produced by cleavage of the polyprotein encoded by ORF1ab. In the ‘subsets’ columns, mutations shared among multiple subsets deriving from the same clade are indicated with a ‘C’, while those subset-specific are reported as ‘B’ or ‘b’, depending on whether their frequency within the subset is greater or less than 80%.

**Table 3:** Impact of mutations on protein conformation and stability.

	Gene	Protein/Peptide	Nucleotide	AA Change	Interactions	Stability (ΔΔG)	Structure
S1	ORF1ab	nsp3	7767	I1683T	Decreased hydrophobic interactions to V1678, L1685		I-Tasser model
		helicase	17,104	H290Y	Hbond to peptide bond of E261. Stacking with F262	Stabilizing ++ (+2.790 kcal/mol)	5RL9
	S	spike	22,879	N439K			
S2–3	ORF1ab	2’-o-ribose methyl transferase	21,154	S166A	Hydrophobic interactions to L59 and L126 (H bond to N210 abolished)	Stabilizing + (+0.425 kcal/mol)	6W75
S4	ORF1ab	nsp3	3267	T183I	Remove a OH group. No polar interaction to Q185 or Q180.		I-Tasser model
			5388	A890D	No specific interaction. At the N-terminal of an a-helix		
			6954	I1412T	Smaller side chain. No specific interaction		
		nsp6	11,288–11,290	S106-			
			11,291–11,293	G107-			
			11,294–11,296	F108-			
		helicase	17,615	K460R	Hbond to Y457 and electrostatic interaction to D458. Contact to F437	Stabilizing ++ (+1.254 kcal/mol)	5RL9
	S	spike	21,767–21,769	H69-			
			21,770	V70-			
			21,992–21,993	Y144-			
			23,063	N501Y			
			23,271	A570D			
			23,604	P681H			
			23,709	T716I			
			24,506	S982A	Loss of an inter-protomer H-bond between the S982 and T547 side chains D1118H: S2		
			24,914	D1118H			
	ORF8	ORF8	27,972	Q27 *			7JX6
			28,048	R52I	Solvent exposed. Contact to S54		
			28,111	Y73C	Solvent exposed		
	N	nucleocapside	28,280–28,282	D3L	Hydrophobic interactions to V324		
			28,977	S235F	Exposed at the N-terminus of an a-helix		
S5	N	nucleocapside	28,487	V72I	Increases hydrophobic interactions		
			28,868	P199S	H-bond to S197		
S7	ORF1ab	nsp4	9526	M324I	Hydrophobic interactions to L321, L323 of the opposite helix		I-Tasser model
		RdRp	13,993	A185S	Add H-bond to V182 and N213 peptide bond		6YYT
			15,766	V776L	Increases hydrophobic interactions to H752, F753 and Y748		
		helicase	16,889	K218R	Exposed to the solvent		5RL9
			17,019	E261D	Hbond to S259 (H to Y324 and H290 are abolished)		
	S	spike	22,992	S477N			
	ORF3a	ORF3a	25,563	Q57H	Contact to His57 from the other subunit. Wall of the central pore. Interacts to Lys61	Stabilizing ++ (+1.620 kcal/mol)	6XDC
	N	nucleocapside	28,975	M234I	C-terminal of an a-helix		
			29,399	A376T	Potential Hbond to K374		
S8	ORF1ab	nsp6	11,132	A3623S			
	S	spike	23,587	Q675H			
	ORF8	ORF8	27,982	P30L	Solvent exposed	Stabilizing ++ (+1.620 kcal/mol)	7JX6
S10	ORF1ab	nsp3	7086	T1456I	Exposed in a loop at the C-terminal of an a-helix. Removes polar interaction to N1457		I-Tasser model
	S	spike	23,477	G639S			
	N	nucleocapside	29,366	P365S	Exposed in a loop. Increases local flexibility?		

List of mutations organized by subset and, on a second level, by subgenomic mRNA/ORF. Mutation are expressed as already described in Table 2. The prediction of protein stability (produced using as template the structures indicated in ‘Structure’) is reported in ‘Stability’, where ‘stabilizing’ indicates that the structure of the protein is expected to increase in stability following the corresponding mutation. ‘Interactions’ reports the potential effect of amino acid changes on interaction with neighboring sites. ‘*’ indicate stop codons.

## Data Availability

This study is based on sequence data deposited in the GISAID repository and made accessible through their website. The main results reported in figures and discussed in the text are provided in more detail in the Supplementary Materials.

## Supplementary Materials

The following supporting information can be downloaded at: https://www.mdpi.com/article/10.3390/ijms23084155/s1.

## Code Availability Statement

The software used for this work is mentioned in the Methods section and elsewhere in the article. It is mainly software that has already been made publicly available by other authors. The procedures used for scanning the tree and comparing subsets were specifically developed for this study and used in combination with the other tools as described in the ‘Methods’ section. Pipeline parameter, rule sets, algorithms and code used to apply the described procedures to the presented data are provided in the ‘Methods’ section, as Supplementary Material or available on request.
