# Supplementary material for: SARS-CoV-2 Pandemic Tracing in Italy Highlights Lineages with Mutational Burden in Growing Subsets
Source: Int J Mol Sci. 2022 Apr 9;23(8):4155. doi: 10.3390/ijms23084155 (PMC9029933; doi:10.3390/ijms23084155)
Supplement: Supplementary file 1 [file ijms-23-04155-s001.zip › Supplementary material.pdf]

International Journal of *Molecular Sciences*

MDPI

Article

SARS-Cov-2 Pandemic Tracing in Italy Highlights Lineages with Mutational Burden in Growing Subsets

Angelo Boccia [1], Rossella Tufano [1], Veronica Ferrucci [1,2], Leandra Sepe [1,2], Martina Bianchi [3], Stefano Pascarella [3], Massimo Zollo [1,2,4,*] and Giovanni Paolella [1,2,*]

[1] CEINGE Biotecnologie Avanzate, Naples, 80145, Italy;
[2] Dipartimento di Medicina Molecolare e Biotecnologie Mediche (DMMBM), "Federico II" University of Naples, Naples, 80131, Italy;
[3] Sapienza Università di Roma, Department of Biochemical Sciences "A. Rossi Fanelli", Rome 00185, Italy;
[4] DAI Medicina di Laboratorio e Trasfusionale, AOU Azienda Ospedaliera 'Federico II', Naples, 80131, Italy;
* Correspondence: GP giovanni.paolella@unina.it; MZ massimo.zollo@unina.it

Citation: Boccia, A.; Tufano, R.; Ferrucci, V.; Sepe, L.; Bianchi, M.; Pascarella, S.; Zollo, M.; Paolella, G. SARS-CoV-2 Pandemic Tracing in Italy Highlights Lineages with Mutational Burden in Growing Subsets. *Int. J. Mol. Sci.* **2022**, *23*, 4155. https://doi.org/10.3390/ijms23084155

Academic Editor: Chih-Jen Yang

Received: 2 March 2022
Accepted: 6 April 2022
Published: 9 April 2022

Publisher's Note: MDPI stays neutral with regard to jurisdictional claims in published maps and institutional affiliations.

Copyright: © 2022 by the authors. Licensee MDPI, Basel, Switzerland. This article is an open access article distributed under the terms and conditions of the Creative Commons Attribution (CC BY) license (https://creativecommons.org/licenses/by/4.0/).

Table S1. Relationship between subsets and Nextstrain clades.

	19A	19B	20A	20A.EU2	20A N.194L	20B	20B S.484K	20C	20D	20E (EU1)	20F	20G	20H 501Y.V2	20I 501Y.V1
**S01**	0	0	0,05	0	0	0	0	0	0	0	0	0	0	0
**S02**	0	0	0	0	0	0	0	0	0,35	0	0	0	0	0
**S03**	0	0	0	0	0	0	0	0	0,68	0	0	0	0	0
**S04**	0	0	0	0	0	0	0	0	0	0	0	0	0	1
**S05**	0	0	0	0	0	0,04	0	0	0	0	0	0	0	0
**S06**	0	0	0	0,19	0	0	0	0	0	0	0	0	0	0
**S07**	0	0	0	1	0	0	0	0	0	0	0	0	0	0
**S08**	0	0	0	0	0	0	0	0	0	0,12	0	0	0	0
**S09**	0	0	0	0	0	0	0	0	0	0,08	0	0	0	0
**S10**	0	0	0	0	0	0	0	0	0	0,12	0	0	0	0

The number reported in each cell indicates the degree of overlap between subset and clade, with '1' representing a perfect match and '0' no overlap.

Table S2. Multiple alignment of the consensus sequence of each subset with major clades.

External file: Table S2.pdf

For the sole positions changed in at least one consensus, compared to the reference one (Wuhan-Hu-1, Refseq NC_045512.2), either the original or the mutated base are reported. The affected mRNA/ORF codon(s) and the genomic coordinates are indicated in the header. For each position both nucleotides and the corresponding amino acid (in brackets) are reported. Sites showing in the subsets a synonymous or non-synonymous change, compared with parent clades, are indicated in light and dark yellow, respectively.

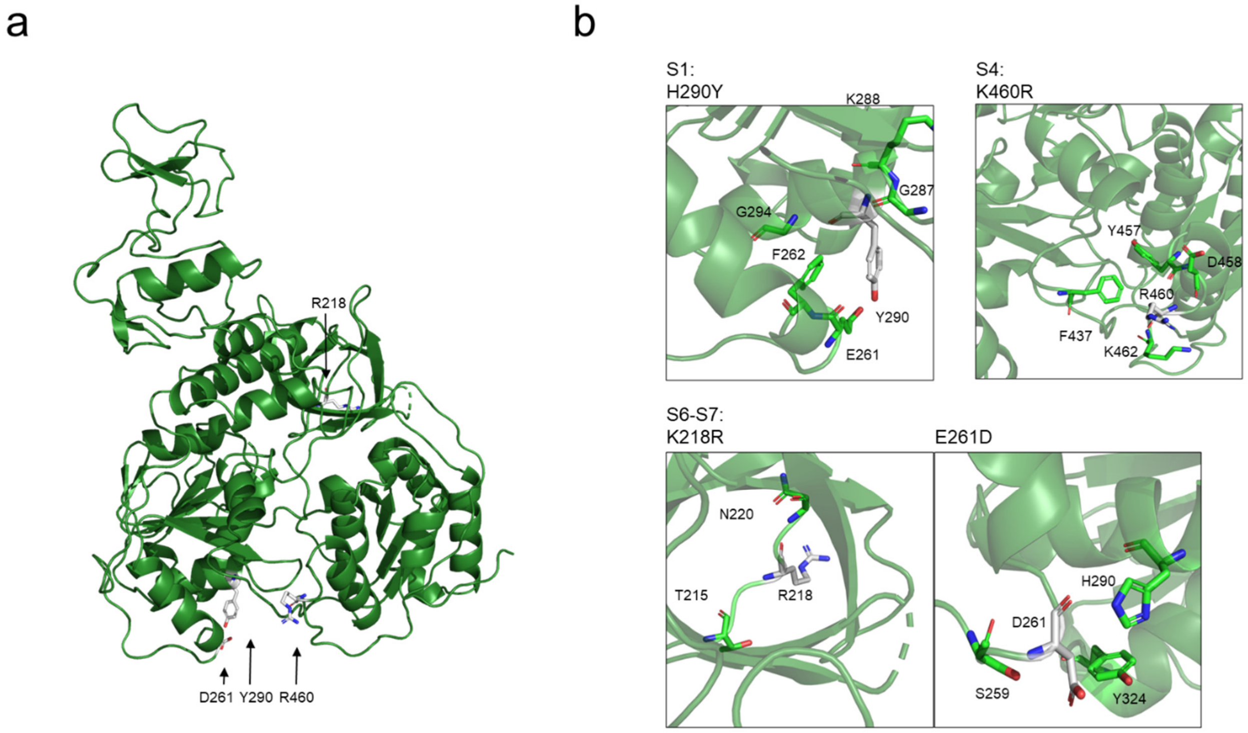

Figure S1. Structural analysis of NSP13 protein. a) Cartoon representation of the protein. b) Residues located in proximity of mutated site are displayed as sticks and labelled.

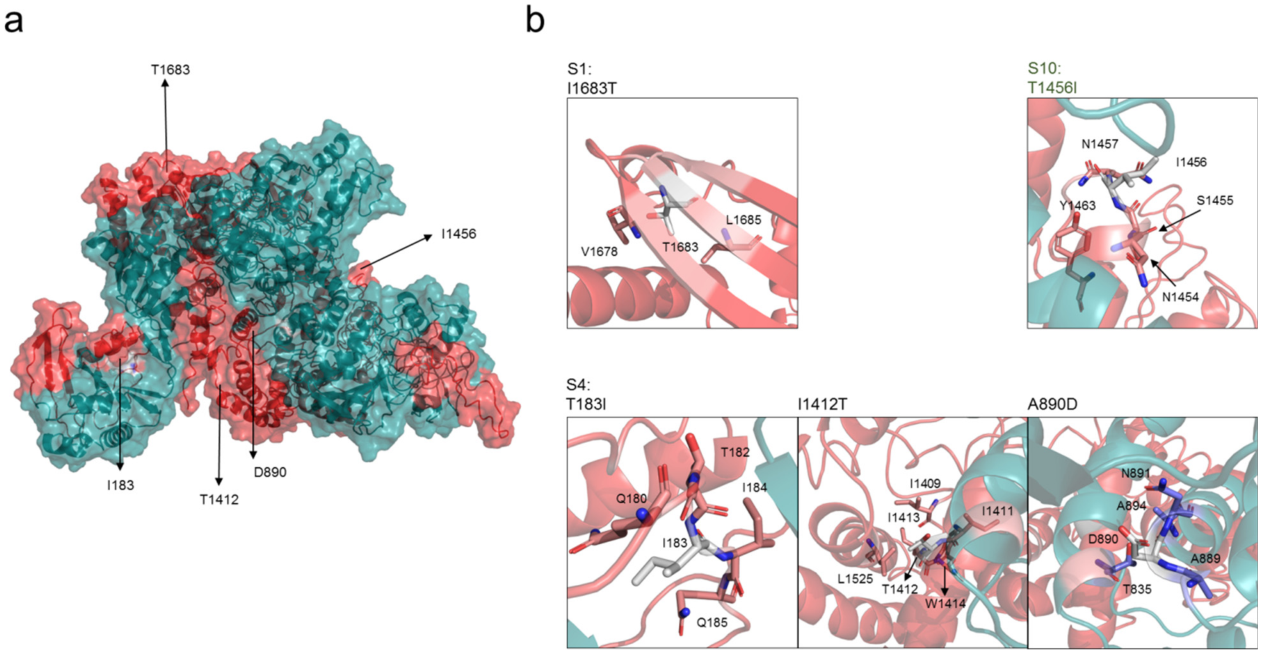

Figure S2. Structural analysis of NSP3 protein. a) Surface representation of SARS-CoV-2 NSP3 model, structured domains are shown in sky-blue and unstructured domains are shown in red. b) The mutated sites are highlighted in the black box Residues located in proximity of mutated site are displayed as sticks and labelled.

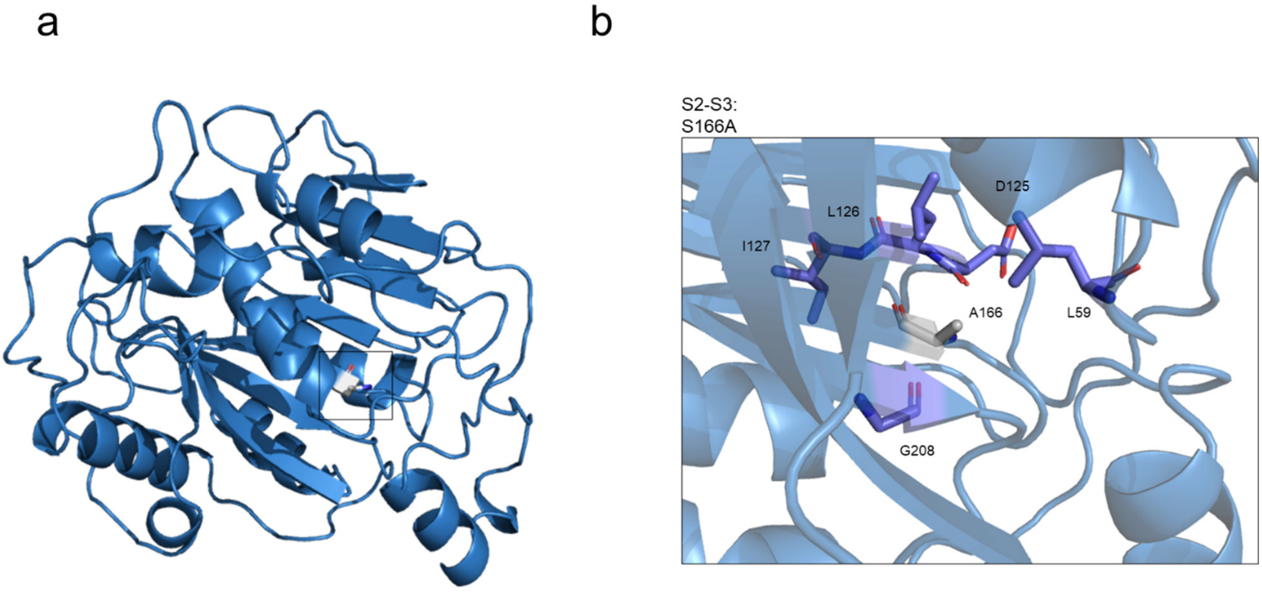

Figure S3. Structural analysis of NSP16 protein. a) Cartoon representation of the SARS-CoV-2 NSP16. The mutated site is highlighted in the black box. b) Residues located in proximity of mutated site are displayed as sticks and labelled.

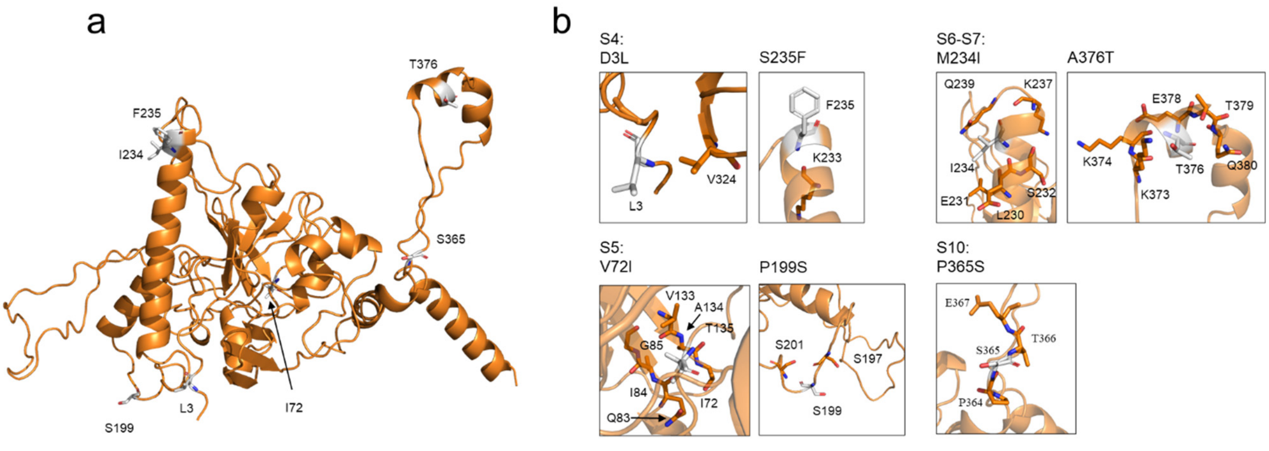

Figure S4. Structural analysis of N protein. a) Three-dimensional model. b) Residues located in proximity of mutated site are displayed as sticks and labelled.

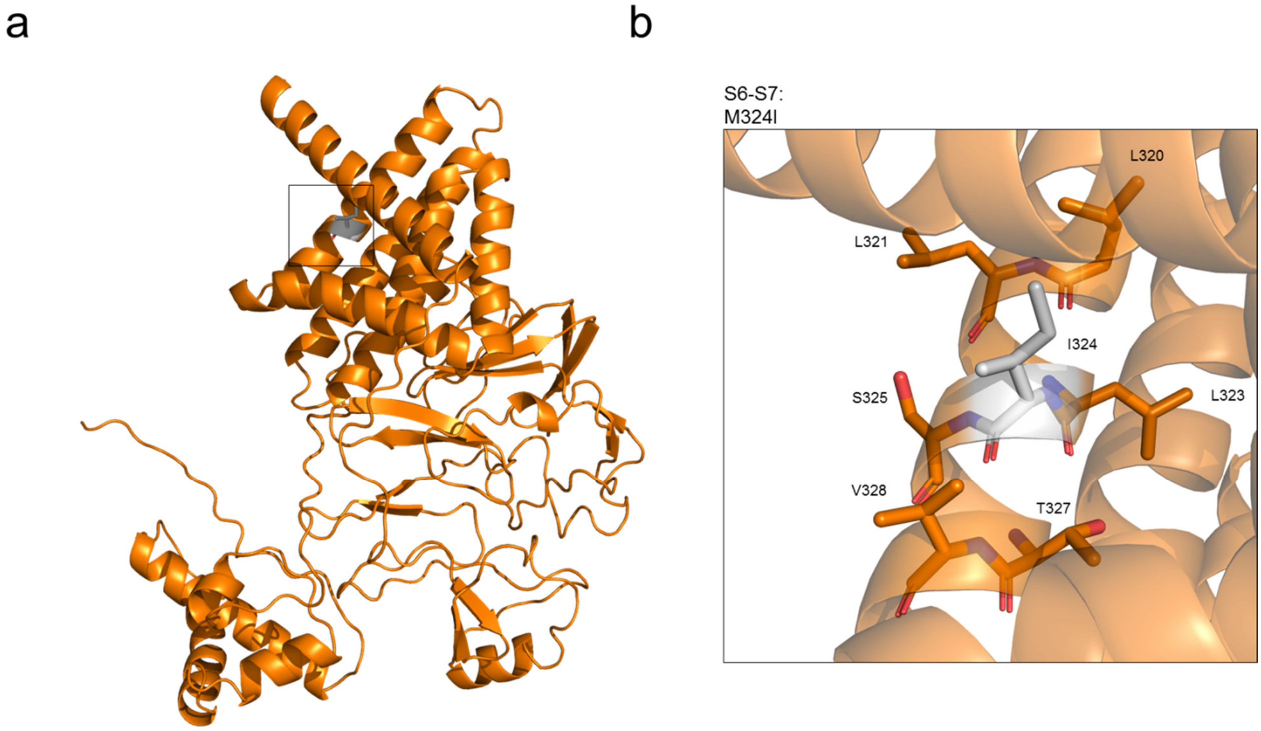

Figure S5. Structural analysis of NSP4 protein. a) Cartoon representation of the protein; the mutated site is highlighted in the black box. b) Detail of I324, corresponding to the mutated site. Residues located in proximity of mutated site are displayed as sticks and labelled.

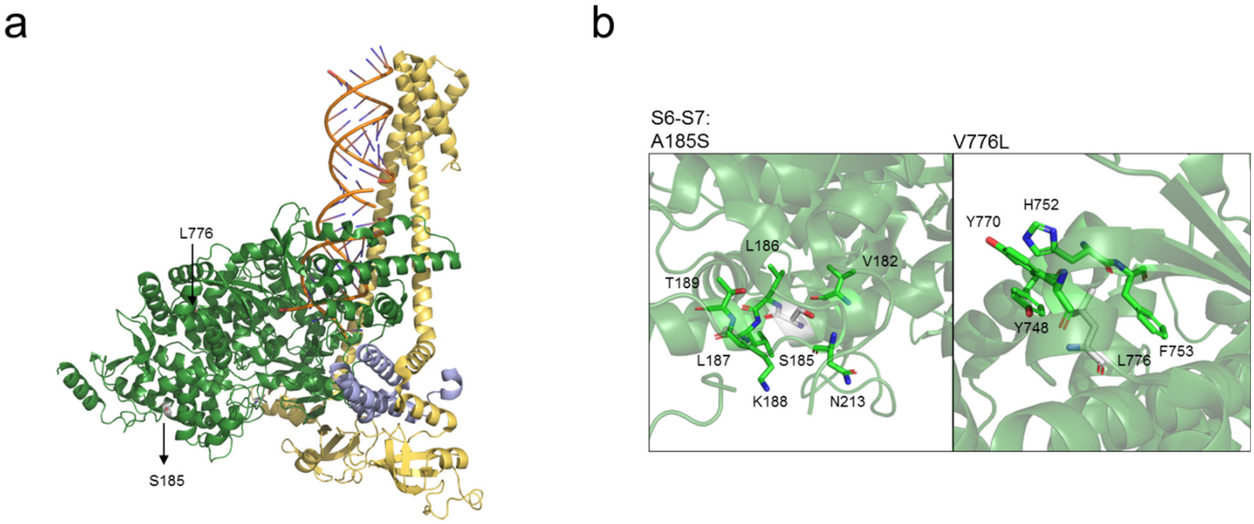

Figure S6. Structural analysis of NSP12 protein. a) Structure of the replicating SARS-CoV-2 polymerase. NSP8 subunits are represented as yellow cartoon. NSP7 and NSP12 as light blue and green cartoon respectively. RNA is in orange. b) Detail of the sites containing the mutant residues.

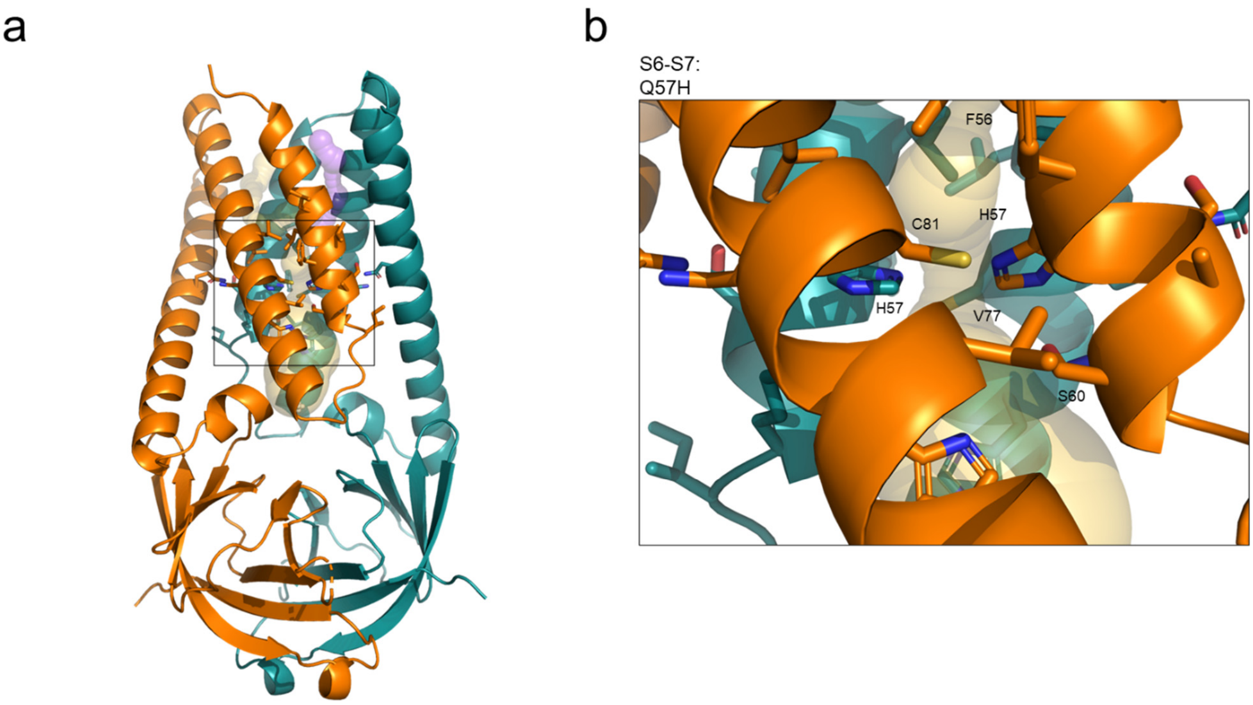

Figure S7. Structural analysis of ORF3a dimer. a) The two subunits are represented as orange and deep teal ribbons. b) Detail of the mutant site and surrounding residues. Residues are displayed as labelled sticks. Transparent yellow spheres delimit part of the central pore. The dimer is represented with the two-fold axis of symmetry approximately parallel to the sheet plane.

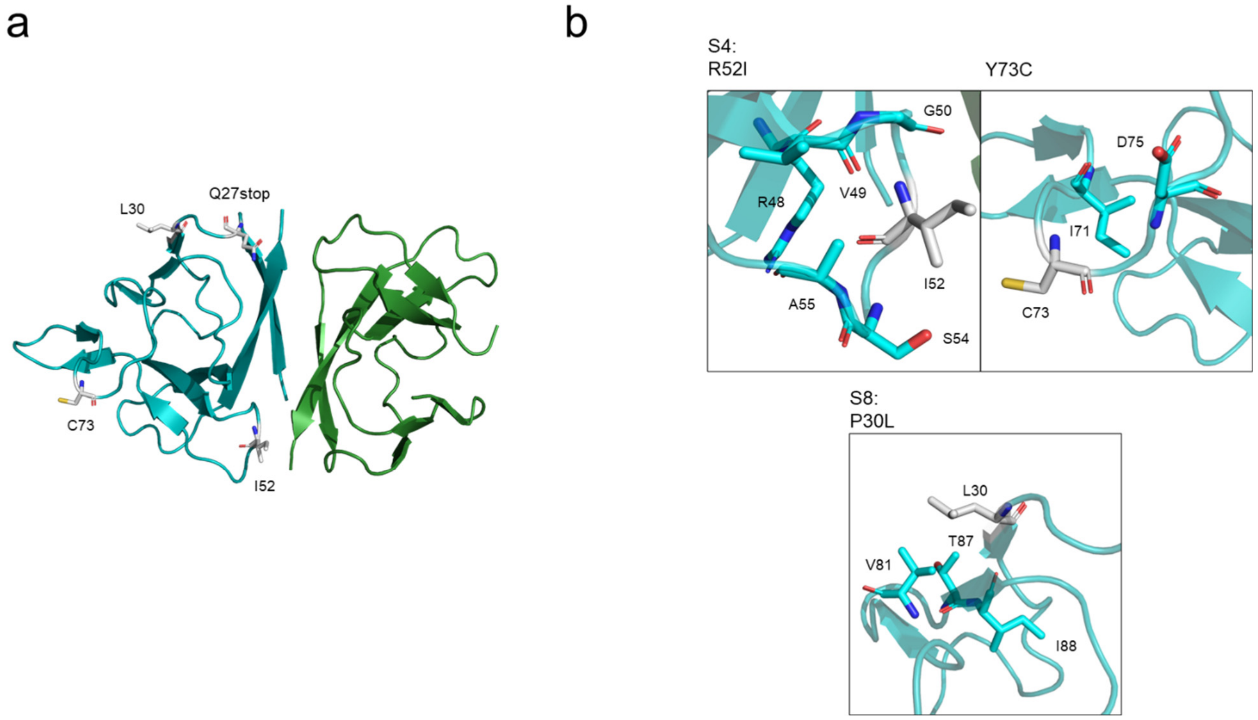

Figure S8. Structural analysis of ORF8 protein. a) Cartoon representation of the SARS-CoV-2 ORF8. The two monomers are displayed in green and cyan, respectively. b) Residues located in proximity of mutated site are displayed as sticks and labelled.

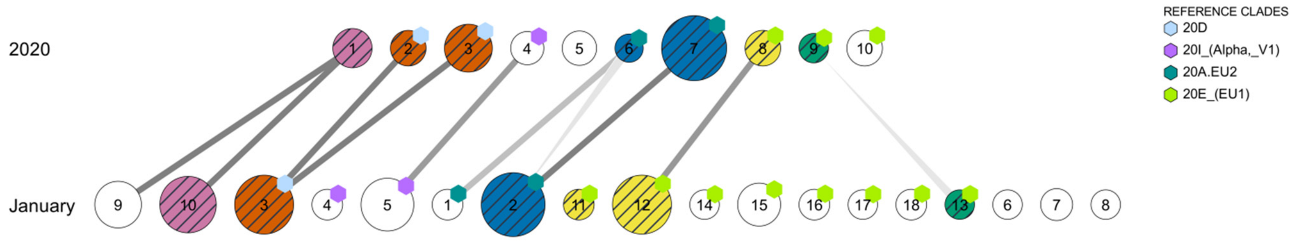

Figure S9. Comparison of '2020' and 'January' subsets. The figure shows the relationships between subsets identified in the analysis of the '2020' dataset and those derived from the first dataset used for the tracing analysis. See 'Figure 3' for a full description of the elements reported in the figure.

Table S3. Subset relationship with known clades/lineages

Subset	Clade	Pango		Subset	Clade	Pango	
		Assignment	Relation			Assignment	Relation
January				February			
1	20A.EU2	B.1.160	par	1	20A	B.1.416	ovr
2	20A.EU2	B.1.160	ovr	2	20A	B.1.258	par
3	20D	C.18	ovr	3	20A.EU2	B.1.160	con
4	20I (Alpha)	B.1.1.7	par	4	20A.EU2	B.1.160	con
5	20I (Alpha)	B.1.1.7	ovr	5	20A.EU2	B.1.160	par
6	20B	B.1.1.229	con	6	20A	B.1.221	ovr
7	20A	B.1.221	ovr	7	20E (EU1)	B.1.177	par
8	20A	B.1.416	ovr	8	20E (EU1)	B.1.177	par
9	20A	B.1.258.14	sub	9	20E (EU1)	B.1.177	par
10	20A	B.1.258	sub	10	20E (EU1)	B.1.177	par
11	20E (EU1)	B.1.177	par	11	20E (EU1)	B.1.177.75	con
12	20E (EU1)	B.1.177	par	12	20E (EU1)	B.1.177.83	ovr
13	20E (EU1)	B.1.177	par	13	20E (EU1)	B.1.177	par
14	20E (EU1)	B.1.177.75	con	14	20E (EU1)	B.1.177	par
15	20E (EU1)	B.1.177.75	con	15	20D	C.18	ovr
16	20E (EU1)	B.1.177.83	con	16	20D	C.18	sub
17	20E (EU1)	B.1.177	par	17	20I (Alpha)	B.1.1.7	par
18	20E (EU1)	B.1.177	par				
March				April			
1	20H (Beta)	B.1.351	sub	1	19B	A	par
2	20A.EU2	B.1.160	sub	2	20H (Beta)	B.1.351	ovr
3	20A	B.1.258.17	ovr	3	20A.EU2	B.1.160	ovr
4	20A	B.1.258	con	4	20A	B.1.258	con
5	20A	B.1.258	con	5	20A	B.1.258	con
6	20E (EU1)	B.1.177	par	6	20A	B.1.258	con
7	20E (EU1)	B.1.177	par	7	20E (EU1)	B.1.177	par
8	20E (EU1)	B.1.177.75	con	8	20E (EU1)	B.1.177.75	con
9	20E (EU1)	B.1.177	par	9	20E (EU1)	B.1.177	par
10	20D	C.18	sub	10	20J (Gamma)	P.1.1	ovr
11	20J (Gamma)	P.1.1	ovr	11	20I (Alpha)	B.1.1.7	par
12	20I (Alpha)	B.1.1.7	par	12	20I (Alpha)	B.1.1.7	par
13	20I (Alpha)	B.1.1.7	par	13	20I (Alpha)	B.1.1.7	par
14	20I (Alpha)	B.1.1.7	par	14	20I (Alpha)	B.1.1.7	par
15	20I (Alpha)	B.1.1.7	par	15	20I (Alpha)	B.1.1.7	par
16	20I (Alpha)	B.1.1.7	par	16	20I (Alpha)	B.1.1.7	par
17	20I (Alpha)	B.1.1.7	par	17	20I (Alpha)	B.1.1.7	par
				18	20I (Alpha)	B.1.1.7	par
May				June			
1	20A	B.1.258	ovr	1	20H (Beta)	B.1.351	ovr
2	20H (Beta)	B.1.351	ovr	2	21A (Delta)	B.1.617.2	ovr
3	21D (Eta)	B.1.525	ovr	3	21D (Eta)	B.1.525	ovr
4	20J (Gamma)	P.1.1	ovr	4	20I (Alpha)	B.1.1.7	par
5	20I (Alpha)	B.1.1.7	par	5	20I (Alpha)	B.1.1.7	par
6	20I (Alpha)	B.1.1.7	par	6	20I (Alpha)	B.1.1.7	par
7	20I (Alpha)	B.1.1.7	par	7	20I (Alpha)	B.1.1.7	par
8	20I (Alpha)	B.1.1.7	par	8	20I (Alpha)	B.1.1.7	par
9	20I (Alpha)	B.1.1.7	par	9	20I (Alpha)	B.1.1.7	par
10	20I (Alpha)	B.1.1.7	par	10	20I (Alpha)	B.1.1.7	par
11	20I (Alpha)	B.1.1.7	par	11	20I (Alpha)	B.1.1.7	par
12	20I (Alpha)	B.1.1.7	par				
13	20I (Alpha)	B.1.1.7	par				

14	20I (Alpha)	B.1.1.7	par				
15	20I (Alpha)	B.1.1.7	par				
July				August			
1	21A (Delta)	B.1.617.2	par	1	20H (Beta)	B.1.351	sub
2	21A (Delta)	B.1.617.2	par	2	21A (Delta)	B.1.617.2	par
3	21A (Delta)	B.1.617.2	par	3	21A (Delta)	B.1.617.2	par
4	20H (Beta)	B.1.351	par	4	21A (Delta)	B.1.617.2	par
5	21D (Eta)	B.1.525	ovr	5	21A (Delta)	B.1.617.2	par
6	20J (Gamma)	P.1.1	con	6	21D (Eta)	B.1.525	ovr
7	20I (Alpha)	B.1.1.7	par	7	20I (Alpha)	B.1.1.7	par
8	20I (Alpha)	B.1.1.7	par				
9	20I (Alpha)	B.1.1.7	par				
September				October			
1	21A (Delta)	B.1.617.2	par	1	21A (Delta)	B.1.617.2	sub*
2	21A (Delta)	B.1.617.2	par	2	21A (Delta)	AY.122	con*
3	21A (Delta)	B.1.617.2	par	3	21A (Delta)	AY.102	sub*
4	21A (Delta)	B.1.617.2	par	4	21A (Delta)	AY.43	con
				5	21A (Delta)	AY.43	con
				6	21A (Delta)	AY.43	con
November				December			
1	20J (Gamma)	P.1.1	con	1	21A (Delta)	AY.9.2	ovr
2	21A (Delta)	B.1.617.2	par	2	21A (Delta)	AY.122	con
3	21A (Delta)	B.1.617.2	par*	3	21A (Delta)	AY.43	con
4	21A (Delta)	AY.43	con	4	21A (Delta)	AY.4	con
5	21A (Delta)	AY.42	ovr	5	21A (Delta)	AY.4	con
6	21A (Delta)	AY.4	con	6	21A (Delta)	AY.46.6	ovr
7	21A (Delta)	AY.4	con	7	21A (Delta)	AY.98.1	ovr
8	21A (Delta)	AY.122	con	8	21A (Delta)	AY.122	con
9	21A (Delta)	AY.98.1	ovr				

The analysis covers all subsets identified in 2021, organized by dataset. For each subset the closest Nextrain clade and Pango lineage are indicated. The relation with the Pango lineage was evaluated by using the official pangolin tool, as described under 'Materials and Methods', and annotated as follows:
[ovr] Pango lineage overlaps with the subset;
[con] Pango lineage contains the subset;
[par] Pango lineage is coincident with the Nextstrain parental clade from which the subset is derived;
[sub] Pango lineage is a subpart of the subset.

Table S4. GISAID acknowledgments.

External file: Table S4.xlsx

List of analyses GISAID sequences grouped by dataset, provided with authors and originating and submitting laboratory.

Supplementary File 1. Sampling rules for the 2020 dataset

External file: builds_2020.yaml

Sampling rules used in the Nextstrain/ncov workflow to analyze the 2020 dataset.

Supplementary File 2. Sampling rules for 2021 datasets

External file: builds_2021.yaml

Sampling rules used in the Nextstrain/ncov workflow to analyze the 2021 datasets.

Supplementary File 3. Nextstrain clades signatures

External file: clade_signatures.txt
