# Supplementary material for: SARS-CoV-2 Pandemic Tracing in Italy Highlights Lineages with Mutational Burden in Growing Subsets
Source: Int J Mol Sci. 2022 Apr 9;23(8):4155. doi: 10.3390/ijms23084155 (PMC9029933; doi:10.3390/ijms23084155)
Supplement: Supplementary file 1 [file ijms-23-04155-s001.zip › Table S2.pdf]

Genome pos.	241	445	913	1059	3037	3267	4002	4543	5170	5388	5629	5986	6286	6954	7086	7767	8047	8140	8782	9130	9526	10097	10252
Region	5UTR	ORF1ab																					
Region pos.	241	60	216	265	924	1001	1246	1426	1635	1708	1788	1907	2007	2230	2274	2501	2594	2625	2839	2955	3087	3278	3329
Peptide		Leader	Nsp2		Nsp3														Nsp4			3C-like proteinase	
Peptide pos.		60	36	85	106	183	428	608	817	890	970	1089	1189	1412	1456	1683	1776	1807	76	192	324	15	66
19A	C	T (V)	C (S)	C (T)	C (F)	C (T)	C (T)	C (T)	C (Y)	C (A)	G (T)	C (F)	C (T)	T (I)	C (T)	T (I)	C (Y)	C (S)	C (S)	G (V)	G (M)	G (G)	C (F)
19B	C	T (V)	C (S)	C (T)	C (F)	C (T)	C (T)	C (T)	C (Y)	C (A)	G (T)	C (F)	C (T)	T (I)	C (T)	T (I)	C (Y)	C (S)	T (S)	G (V)	G (M)	G (G)	C (F)
20A	T	T (V)	C (S)	C (T)	T (F)	C (T)	C (T)	C (T)	C (Y)	C (A)	G (T)	C (F)	C (T)	T (I)	C (T)	T (I)	C (Y)	C (S)	C (S)	G (V)	G (M)	G (G)	C (F)
S01	T	T (V)	C (S)	C (T)	T (F)	C (T)	C (T)	C (T)	C (Y)	C (A)	G (T)	C (F)	C (T)	T (I)	C (T)	C (T)	T (Y)	C (S)	C (S)	G (V)	G (M)	G (G)	C (F)
S06	T	T (V)	C (S)	C (T)	T (F)	C (T)	C (T)	T (T)	C (Y)	C (A)	T (T)	C (F)	C (T)	T (I)	C (T)	T (I)	C (Y)	C (S)	C (S)	G (V)	T (I)	G (G)	C (F)
S07	T	T (V)	C (S)	C (T)	T (F)	C (T)	C (T)	T (T)	C (Y)	C (A)	T (T)	C (F)	C (T)	T (I)	C (T)	T (I)	C (Y)	C (S)	C (S)	G (V)	T (I)	G (G)	C (F)
S08	T	C (V)	C (S)	C (T)	T (F)	C (T)	C (T)	C (T)	T (Y)	C (A)	G (T)	C (F)	T (T)	T (I)	C (T)	T (I)	C (Y)	T (S)	C (S)	G (V)	G (M)	G (G)	C (F)
S09	T	C (V)	C (S)	C (T)	T (F)	C (T)	C (T)	C (T)	C (Y)	C (A)	G (T)	C (F)	T (T)	T (I)	C (T)	T (I)	C (Y)	C (S)	C (S)	G (V)	G (M)	G (G)	C (F)
S10	T	C (V)	C (S)	C (T)	T (F)	C (T)	C (T)	C (T)	C (Y)	C (A)	G (T)	C (F)	T (T)	T (I)	T (I)	T (I)	C (Y)	C (S)	C (S)	G (V)	G (M)	G (G)	C (F)
20B	T	T (V)	C (S)	C (T)	T (F)	C (T)	C (T)	C (T)	C (Y)	C (A)	G (T)	C (F)	C (T)	T (I)	C (T)	T (I)	C (Y)	C (S)	C (S)	G (V)	G (M)	G (G)	C (F)
S02	T	T (V)	C (S)	C (T)	T (F)	C (T)	T (I)	C (T)	C (Y)	C (A)	G (T)	C (F)	C (T)	T (I)	C (T)	T (I)	C (Y)	C (S)	C (S)	G (V)	G (M)	A (S)	T (F)
S03	T	T (V)	C (S)	C (T)	T (F)	C (T)	T (I)	C (T)	C (Y)	C (A)	G (T)	C (F)	C (T)	T (I)	C (T)	T (I)	C (Y)	C (S)	C (S)	G (V)	G (M)	A (S)	T (F)
S04	T	T (V)	T (S)	C (T)	T (F)	T (I)	C (T)	C (T)	C (Y)	A (D)	G (T)	T (F)	C (T)	C (T)	C (T)	T (I)	C (Y)	C (S)	C (S)	G (V)	G (M)	G (G)	C (F)
S05	T	T (V)	C (S)	C (T)	T (F)	C (T)	C (T)	C (T)	C (Y)	C (A)	G (T)	C (F)	C (T)	T (I)	C (T)	T (I)	C (Y)	C (S)	C (S)	T (V)	G (M)	G (G)	C (F)
20C	T	T (V)	C (S)	T (I)	T (F)	C (T)	C (T)	C (T)	C (Y)	C (A)	G (T)	C (F)	C (T)	T (I)	C (T)	T (I)	C (Y)	C (S)	C (S)	G (V)	G (M)	G (G)	C (F)

Genome pos.	11132	288-112	291-112	294-112	11497	11956	13536	13993	14408	14676	15279	15766	16111	16176	16889	17019	17104	17615	18877	20268	21138	21154	21255
Region	ORF1ab																						
Region pos.	3623	3675	3676	3677	3744	3897	4424	4577	4715	4804	5005	5168	5283	5304	5542	5585	5614	5784	6205	6668	6958	6964	6997
Peptide	Nsp6					Nsp7	RdRp								Helicase				3’-to-5’ exon.	endoR NAse	2’-o-MT		
Peptide pos.	54	106	107	108	175	38	32	185	323	412	613	776	891	912	218	261	290	460	280	216	160	166	199
19A	G (A)	TCT (S)	GGT (G)	TTT (F)	C (Y)	C (D)	C (Y)	G (A)	C (P)	C (P)	C (H)	G (V)	C (L)	T (T)	A (K)	G (E)	C (H)	A (K)	C (L)	A (L)	G (K)	T (S)	G (A)
19B	G (A)	TCT (S)	GGT (G)	TTT (F)	C (Y)	C (D)	C (Y)	G (A)	C (P)	C (P)	C (H)	G (V)	C (L)	T (T)	A (K)	G (E)	C (H)	A (K)	C (L)	A (L)	G (K)	T (S)	G (A)
20A	G (A)	TCT (S)	GGT (G)	TTT (F)	C (Y)	C (D)	C (Y)	G (A)	T (L)	C (P)	C (H)	G (V)	C (L)	T (T)	A (K)	G (E)	C (H)	A (K)	C (L)	A (L)	G (K)	T (S)	G (A)
S01	G (A)	TCT (S)	GGT (G)	TTT (F)	C (Y)	C (D)	C (Y)	G (A)	T (L)	C (P)	C (H)	G (V)	C (L)	T (T)	A (K)	G (E)	T (Y)	A (K)	C (L)	G (L)	G (K)	T (S)	G (A)
S06	G (A)	TCT (S)	GGT (G)	TTT (F)	T (Y)	C (D)	C (Y)	T (S)	T (L)	C (P)	C (H)	T (L)	C (L)	T (T)	G (R)	T (D)	C (H)	A (K)	T (L)	A (L)	G (K)	T (S)	G (A)
S07	G (A)	TCT (S)	GGT (G)	TTT (F)	T (Y)	C (D)	C (Y)	T (S)	T (L)	C (P)	C (H)	T (L)	C (L)	T (T)	G (R)	T (D)	C (H)	A (K)	T (L)	A (L)	G (K)	T (S)	G (A)
S08	T (S)	TCT (S)	GGT (G)	TTT (F)	C (Y)	C (D)	C (Y)	G (A)	T (L)	C (P)	C (H)	G (V)	C (L)	T (T)	A (K)	G (E)	C (H)	A (K)	C (L)	A (L)	G (K)	T (S)	C (A)
S09	G (A)	TCT (S)	GGT (G)	TTT (F)	C (Y)	C (D)	C (Y)	G (A)	T (L)	C (P)	C (H)	G (V)	C (L)	T (T)	A (K)	G (E)	C (H)	A (K)	C (L)	A (L)	G (K)	T (S)	C (A)
S10	G (A)	TCT (S)	GGT (G)	TTT (F)	C (Y)	C (D)	C (Y)	G (A)	T (L)	C (P)	C (H)	G (V)	C (L)	T (T)	A (K)	G (E)	C (H)	A (K)	C (L)	A (L)	G (K)	T (S)	C (A)
20B	G (A)	TCT (S)	GGT (G)	TTT (F)	C (Y)	C (D)	C (Y)	G (A)	T (L)	C (P)	C (H)	G (V)	C (L)	T (T)	A (K)	G (E)	C (H)	A (K)	C (L)	A (L)	G (K)	T (S)	G (A)
S02	G (A)	TCT (S)	GGT (G)	TTT (F)	C (Y)	T (D)	T (Y)	G (A)	T (L)	C (P)	C (H)	G (V)	C (L)	T (T)	A (K)	G (E)	C (H)	A (K)	C (L)	A (L)	A (K)	G (A)	G (A)
S03	G (A)	TCT (S)	GGT (G)	TTT (F)	C (Y)	T (D)	T (Y)	G (A)	T (L)	C (P)	C (H)	G (V)	C (L)	T (T)	A (K)	G (E)	C (H)	A (K)	C (L)	A (L)	A (K)	G (A)	G (A)
S04	G (A)	--- (-)	--- (-)	--- (-)	C (Y)	C (D)	C (Y)	G (A)	T (L)	T (P)	T (H)	G (V)	C (L)	C (T)	A (K)	G (E)	C (H)	G (R)	C (L)	A (L)	G (K)	T (S)	G (A)
S05	G (A)	TCT (S)	GGT (G)	TTT (F)	C (Y)	C (D)	C (Y)	G (A)	T (L)	C (P)	C (H)	G (V)	T (L)	T (T)	A (K)	G (E)	C (H)	A (K)	C (L)	A (L)	G (K)	T (S)	G (A)
20C	G (A)	TCT (S)	GGT (G)	TTT (F)	C (Y)	C (D)	C (Y)	G (A)	T (L)	C (P)	C (H)	G (V)	C (L)	T (T)	A (K)	G (E)	C (H)	A (K)	C (L)	A (L)	G (K)	T (S)	G (A)

Genome pos.	21765 -21766	21767 -21769	21770	21991	992-219	22227	22879	22992	23063	23271	23403	23477	23587	23604	23709	23731	24506	24914	25563	25710	26735	26801	26876
Region	S																		ORF3a		M		
Region pos.	68	69	70	143	144	222	439	477	501	570	614	639	675	681	716	723	982	1118	57	106	71	93	118
Peptide																							
Peptide pos.																							
19A	ATA (I)	CAT (H)	G (V)	T (V)	TAT (Y)	C (A)	C (N)	G (S)	A (N)	C (A)	A (D)	G (G)	G (Q)	C (P)	C (T)	C (T)	T (S)	G (D)	G (Q)	C (L)	C (Y)	C (L)	T (I)
19B	ATA (I)	CAT (H)	G (V)	T (V)	TAT (Y)	C (A)	C (N)	G (S)	A (N)	C (A)	A (D)	G (G)	G (Q)	C (P)	C (T)	C (T)	T (S)	G (D)	G (Q)	C (L)	C (Y)	C (L)	T (I)
20A	ATA (I)	CAT (H)	G (V)	T (V)	TAT (Y)	C (A)	C (N)	G (S)	A (N)	C (A)	G (G)	G (G)	G (Q)	C (P)	C (T)	C (T)	T (S)	G (D)	G (Q)	C (L)	C (Y)	C (L)	T (I)
S01	ATA (I)	CAT (H)	G (V)	T (V)	TAT (Y)	C (A)	A (K)	G (S)	A (N)	C (A)	G (G)	G (G)	G (Q)	C (P)	C (T)	C (T)	T (S)	G (D)	G (Q)	C (L)	C (Y)	C (L)	T (I)
S06	ATA (I)	CAT (H)	G (V)	T (V)	TAT (Y)	C (A)	C (N)	A (N)	A (N)	C (A)	G (G)	G (G)	G (Q)	C (P)	C (T)	C (T)	T (S)	G (D)	T (H)	T (L)	T (Y)	C (L)	C (I)
S07	ATA (I)	CAT (H)	G (V)	T (V)	TAT (Y)	C (A)	C (N)	A (N)	A (N)	C (A)	G (G)	G (G)	G (Q)	C (P)	C (T)	C (T)	T (S)	G (D)	T (H)	T (L)	T (Y)	C (L)	C (I)
S08	ATA (I)	CAT (H)	G (V)	T (V)	TAT (Y)	T (V)	C (N)	G (S)	A (N)	C (A)	G (G)	G (G)	T (H)	C (P)	C (T)	C (T)	T (S)	G (D)	G (Q)	C (L)	C (Y)	G (L)	T (I)
S09	ATA (I)	CAT (H)	G (V)	T (V)	TAT (Y)	T (V)	C (N)	G (S)	A (N)	C (A)	G (G)	G (G)	G (Q)	C (P)	C (T)	C (T)	T (S)	G (D)	G (Q)	C (L)	C (Y)	G (L)	T (I)
S10	ATA (I)	CAT (H)	G (V)	T (V)	TAT (Y)	T (V)	C (N)	G (S)	A (N)	C (A)	G (G)	A (S)	G (Q)	C (P)	C (T)	C (T)	T (S)	G (D)	G (Q)	C (L)	C (Y)	G (L)	T (I)
20B	ATA (I)	CAT (H)	G (V)	T (V)	TAT (Y)	C (A)	C (N)	G (S)	A (N)	C (A)	G (G)	G (G)	G (Q)	C (P)	C (T)	C (T)	T (S)	G (D)	G (Q)	C (L)	C (Y)	C (L)	T (I)
S02	ATA (I)	CAT (H)	G (V)	T (V)	TAT (Y)	C (A)	C (N)	G (S)	A (N)	C (A)	G (G)	G (G)	G (Q)	C (P)	C (T)	T (T)	T (S)	G (D)	G (Q)	C (L)	C (Y)	C (L)	T (I)
S03	ATA (I)	CAT (H)	G (V)	T (V)	TAT (Y)	C (A)	C (N)	G (S)	A (N)	C (A)	G (G)	G (G)	G (Q)	C (P)	C (T)	T (T)	T (S)	G (D)	G (Q)	C (L)	C (Y)	C (L)	T (I)
S04	A-- (-)	--- (-)	- (-)	- (-)	--T (-)	C (A)	C (N)	G (S)	T (Y)	A (D)	G (G)	G (G)	G (Q)	A (H)	T (I)	C (T)	G (A)	C (H)	G (Q)	C (L)	C (Y)	C (L)	T (I)
S05	ATA (I)	CAT (H)	G (V)	T (V)	TAT (Y)	C (A)	C (N)	G (S)	A (N)	C (A)	G (G)	G (G)	G (Q)	C (P)	C (T)	C (T)	T (S)	G (D)	G (Q)	C (L)	C (Y)	C (L)	T (I)
20C	ATA (I)	CAT (H)	G (V)	T (V)	TAT (Y)	C (A)	C (N)	G (S)	A (N)	C (A)	G (G)	G (G)	G (Q)	C (P)	C (T)	C (T)	T (S)	G (D)	T (H)	C (L)	C (Y)	C (L)	T (I)

Genome pos.	27319	27800	27944	27972	27982	28048	28111	28144	28280 -28282	28487	28868	28881 -28882	28883	28932	28975	28977	29366	29399	29645	29706	29734
Region	ORF6	ORF7b	ORF8						ORFN										ORF10	3UTR	
Region pos.	40	15	17	27	30	52	73	84	3	72	199	203	204	220	234	235	365	376	30	32	60
Peptide																					
Peptide pos.																					
19A	T (L)	C (A)	C (H)	C (Q)	C (P)	G (R)	A (Y)	T (L)	GAT (D)	G (V)	C (P)	AGG (R)	G (G)	C (A)	G (M)	C (S)	C (P)	G (A)	G (V)	G	G
19B	T (L)	C (A)	C (H)	C (Q)	C (P)	G (R)	A (Y)	C (S)	GAT (D)	G (V)	C (P)	AGG (R)	G (G)	C (A)	G (M)	C (S)	C (P)	G (A)	G (V)	G	G
20A	T (L)	C (A)	C (H)	C (Q)	C (P)	G (R)	A (Y)	T (L)	GAT (D)	G (V)	C (P)	AGG (R)	G (G)	C (A)	G (M)	C (S)	C (P)	G (A)	G (V)	G	G
S01	T (L)	A (A)	C (H)	C (Q)	C (P)	G (R)	A (Y)	T (L)	GAT (D)	G (V)	C (P)	AGG (R)	G (G)	C (A)	G (M)	C (S)	C (P)	G (A)	G (V)	G	C
S06	T (L)	C (A)	C (H)	C (Q)	C (P)	G (R)	A (Y)	T (L)	GAT (D)	G (V)	C (P)	AGG (R)	G (G)	C (A)	C (I)	C (S)	C (P)	A (T)	G (V)	G	G
S07	T (L)	C (A)	C (H)	C (Q)	C (P)	G (R)	A (Y)	T (L)	GAT (D)	G (V)	C (P)	AGG (R)	G (G)	C (A)	C (I)	C (S)	C (P)	A (T)	G (V)	G	G
S08	T (L)	C (A)	C (H)	C (Q)	T (L)	G (R)	A (Y)	T (L)	GAT (D)	G (V)	C (P)	AGG (R)	G (G)	T (V)	G (M)	C (S)	C (P)	G (A)	T (L)	G	G
S09	T (L)	C (A)	T (H)	C (Q)	C (P)	G (R)	A (Y)	T (L)	GAT (D)	G (V)	C (P)	AGG (R)	G (G)	T (V)	G (M)	C (S)	C (P)	G (A)	T (L)	G	G
S10	T (L)	C (A)	C (H)	C (Q)	C (P)	G (R)	A (Y)	T (L)	GAT (D)	G (V)	C (P)	AGG (R)	G (G)	T (V)	G (M)	C (S)	T (S)	G (A)	T (L)	G	G
20B	T (L)	C (A)	C (H)	C (Q)	C (P)	G (R)	A (Y)	T (L)	GAT (D)	G (V)	C (P)	AAA (K)	C (R)	C (A)	G (M)	C (S)	C (P)	G (A)	G (V)	G	G
S02	T (L)	C (A)	C (H)	C (Q)	C (P)	G (R)	A (Y)	T (L)	GAT (D)	G (V)	C (P)	AAA (K)	C (R)	C (A)	G (M)	C (S)	C (P)	G (A)	G (V)	G	G
S03	T (L)	C (A)	C (H)	C (Q)	C (P)	G (R)	A (Y)	T (L)	GAT (D)	G (V)	C (P)	AAA (K)	C (R)	C (A)	G (M)	C (S)	C (P)	G (A)	G (V)	G	G
S04	T (L)	C (A)	C (H)	T (*)	C (P)	T (I)	G (C)	T (L)	CTA (L)	G (V)	C (P)	AAA (K)	C (R)	C (A)	G (M)	T (F)	C (P)	G (A)	G (V)	G	G
S05	C (L)	C (A)	C (H)	C (Q)	C (P)	G (R)	A (Y)	T (L)	GAT (D)	A (I)	T (S)	AAA (K)	C (R)	C (A)	G (M)	C (S)	C (P)	G (A)	G (V)	T	G
20C	T (L)	C (A)	C (H)	C (Q)	C (P)	G (R)	A (Y)	T (L)	GAT (D)	G (V)	C (P)	AGG (R)	G (G)	C (A)	G (M)	C (S)	C (P)	G (A)	G (V)	G	G
